# Study to Probe Subsistence of Host-Guest Inclusion Complexes of α and β-Cyclodextrins with Biologically Potent Drugs for Safety Regulatory Dischargement

**DOI:** 10.1038/s41598-018-31373-x

**Published:** 2018-08-29

**Authors:** Biplab Rajbanshi, Subhadeep Saha, Koyeli Das, Biraj Kumar Barman, Swarnab Sengupta, Arindam Bhattacharjee, Mahendra Nath Roy

**Affiliations:** 1 Department of Chemistry, University of North Bengal, Darjeeling, 734013 India; 2 Department of Microbiology, University of North Bengal, Darjeeling, 734013 India

## Abstract

Host-guest interaction of two significant drugs, phenylephrine hydrochloride and synephrine with α and β-cyclodextrins were studied systematically. Initially two simple but reliable physicochemical techniques namely conductance and surface tension were employed to find out saturation concentration for the inclusion and its stoichiometry. The obtained 1:1 stoichiometry was further confirmed by two spectrometric methods, UV-Vis study and spectrofluorimetry. Significant shifts in IR stretching frequency also support the inclusion process. Relative stabilities of the inclusion complexes were established by the association constants obtained from UV-Vis spectroscopic measurements, program based mathematical calculation of conductivity data. Calculations of the thermodynamic parameters dictates thermodynamic feasibility of the inclusion process. Spectrofluorometric measurement scaffolds the UV-Vis spectroscopic measurement validating stability of the ICs once again. Mass spectroscopic measurement gives the molecular ion peaks corresponding to the inclusion complex of 1:1 molar ratio of host and guest molecules. The mechanism of inclusion was drawn by ^1^H-NMR and 2D ROESY spectroscopic analysis. Surface texture of the inclusion complexes was studied by SEM. Finally, the cytotoxic activities of the inclusion complexes were analyzed and found, Cell viability also balances for non-toxic behavior of the ICs. Moreover, all the studies reveal the formation of inclusion complexes of two ephedra free, alternatively emerging drugs (after their banned product having ephedra) SNP, PEH with α and β-CD which enriches the drug delivery system with their regulatory release without any chemical modification.

## Introduction

**Figure 1:**
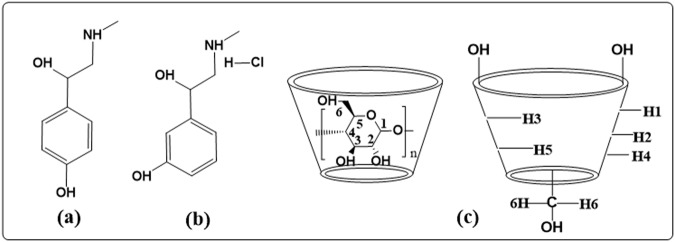
Molecular structures of **(a)** SNP, **(b)** PEH, **(c)** Cyclodextrins, showing the exterior and the interior protons, here, n = 6 to 8 for the α, β and ϒ-cyclodextrins respectively.

In supramolecular chemistry of cyclodextrins various guest molecules having hydrophobic part, influenced by non-covalent interaction, can be inserted into the hydrophobic cavity of cyclodextrin molecules. Cyclodextrins (α-CD, β-CD, γ-CD) having six, seven and eight numbers of glucopyranose units respectively, (Fig. 1) produced from starch by the enzymatic conversion, have different cavity sizes. Inclusion complexes (ICs) with structures of higher complexity in the solid state and solution phase can increase the aqueous solubility of various drugs cum bio-active molecules of merely water solubility which leads to the development of drug delivery systems^1,2^. Chiral Separation of molecules using cyclodextrins as chiral additives are also possible by applying capillary electrophoresis (CE) and electrochemical detection (ED) method^3–6^. Structural characterization of Host-Guest inclusion complexes of α-CD and β-CD with two bio-active molecules, PEH and SNP were done over here in terms of geometry and structural preferences by means of a variety of physical and spectroscopic methods in solid state and solution phase.

Phenylephrine hydrochloride (PEH) (Fig. 1) is a selective α_1_-adrenergic receptor agonist of the phenethylamine class used primarily in cold and flu conditions as an antipyretic, analgesic drug to relief pain^7^. In the United States PEH is used as nasal decongestant. Phenylpropanolamine, pseudoephedrine and ephedrine are also used as nasal decongestant as the substitute of PEH^8,9^. However, due to serious side effect (hemorrhagic stroke) phenylpropanolamine was withdrawn from market^10^. Now it is imperative to find out the suitability of PEH as the same done by the Phenylpropanolamine, pseudoephedrine and ephedrine for the treatment of nasal or sinus congestion and to find out the way of delivery with biocompatibility.

Alkaloid synephrine (SNP) (Fig. 1) was first extracted as a natural product from the leaves of various citrus trees are used as bronchial muscle reluctant, increases blood pressure in the patients suffering from low blood pressure. Its presence and positive retort as a bio-marker makes the orange juice like soft drinks authentic^11^. Lipolytic stimulation by synephrine increases thermogenesis which leads to the increase in metabolic rate and fat oxidation^12–16^. In weight loss products as well as in the dietary supplement “ephedra free” synephrine is frequently used and starts to earn enormous attention after the banned product ephedrine^17,18^. Most of the cases patients suffering from obesity are often found to suffer from type-2 diabetes and hence synephrine in weight loss products frequently becomes beneficial to the diabetic patients^19,20^.

Cyclodextrins, mostly α-cyclodextrin, are found to form complexes with the dietary fat which are stable enough to undergo enzymatic hydrolysis by lipase. This restrains accumulation of fat in human body^21,22^. Hence, inclusion complex of SNP and cyclodextrins can be of a great deal for the weight loss/weight management dietary food supplement for sportsman or obese person^23^.

## Experimental Section

### Materials

Phenylephrine hydrochloride, Synephrine, α and β-cyclodextrin of puris grade of purity ≥98.0% were purchased from Sigma-Aldrich and were kept in a refrigerator as received and used right away.

### Apparatus

Utilizing JASCO V-530 UV−Vis spectrophotometer, UV−visible spectra were recorded with a wavelength accuracy of ±0.5 nm. Cell temperature during the experiment was controlled from 298.15 K to 308.15 K with a digital thermostat.

Studies on surface tension at the experimental temperatures with the accuracy of ±0.1 mN m^−1^ were done by employing K9 digital TENSIOMETER (Krüss GmbH, Hamburg, Germany) which uses the platinum ring detachment technique. The temperature of the experimental solutions was kept constant at 298.15 K by circulating thermostat water through a double-walled glass vessel holding solution.

Proper instrumentation of METTLER-TOLEDO Seven Multi conductivity meter provides specific conductivity values with an uncertainty of ±1.0 μS m^−1^. Constancy in temperature at the specific value of the solutions under experiment was maintained with an auto-thermostatic water bath. HPLC-grade water with a specific conductance of 6.0 μS m^−1^ was utilized for conductivity measurement. Calibration of the Systronics Type CD – 30 conductivity cell was done using 0.01 M freshly prepared aqueous solution of KCl.

Fluorescence spectra were noted via JASCO V-530 UV/VIS Spectrophotometer, at 25 °C in a Hellma quartz cuvette (250–400 nm spectral range, 2.0 mL volume, 10 mm path length) equipped with a magnetic stir bar. To a solution of [SNP/PEH] (100 µM, 1 mL) in deionized water (Millipore, 18.2 MΏ. Cm) was prepared with α-CD and β-CD. (200 µM) in the stock fluorescence spectra were recorded after 1 hr of mixing time. The output range of the machine was nearly about 2 analogs (+/−10 volts).

2D ROESY as well as 1 H NMR spectra were recorded in D_2_O solvent at 400 MHz in Bruker Avance instrument at 298.15 K. The residual protonated signal (HDO, δ 4.79 ppm) was used as an internal standard. The chemical shifts data, δ values are presented in parts per million.

HRMS spectra of the solid ICs were recorded on a quadrupole time-of-flight (Q-TOF) high-resolution instrument with positive-mode electrospray ionization taking the methanol solution of the solid ICs.

FTIR spectral analysis was performed on a Perkin-Elmer FTIR spectrometer in the scanning range of 4000−400 cm^−1^. According to the KBr disk method the disks were made in 1:100 ratios of sample and KBr. Studies were carried out at room temperature and at a humidity of 45%.

SEM: Scanning Electron Microscope (JSM-6360) was aided to perform the analysis and obtain the data’s. It also discusses about the morphological patterns and particle size of the Inclusion Complex.

Antimicrobial activity assay: In this experiment (gram negative *E. coli*), (gram positive B. subtilis) were considered as model organism. This test was done according to the Agar cup method. In brief, spread plate technique was applied to inoculate the organisms in Muller-Hinton agar and the compounds were applied in agar cup at 1 mg/ml concentration in separate plates and incubated at 37 °C for 24 hrs. Double distilled water was used as the control. Antimicrobial activity was determined by means of the zone of inhibition surroundings agar cup. Each of the experiments was done in triplicate.

Cell viability assay: In this experiment pure sample as well as ICs, SNP + α-CD, SNP + β-CD, PEH + α-CD and PEH + β-CD were added in the nutrient agar broth and *E. coli* and B. subtilis were inoculated. After 24 hrs of incubation at 37 °C cells were plated, and colony count was completed. Growth in nutrient broth without the ICs was taken as the control. All the experiment was done in triplicate. Level of significance (p) for all experiment was set to 0.05.

### Procedure

All the solutions under experiment were prepared after checking the solubility of the PEH, SNP and CDs in triply distilled, deionized and degassed water. METTLER TOLEDO AG-285 analytical balance with an uncertainty of ±0.1 mg at 298.15 K was used to weigh all the experimental materials. Loss of materials caused by evaporation during mixing and working with the solutions was minimized by taking sufficient precautions. For the preparation of the solid inclusion complexes, 20 mL 1.0 (mM) solutions of α and β-CD were prepared separately with triply distilled, deionized and degassed water which, allowed to stir for 6 hours on a magnetic stirrer. Then, 20 mL 1.0 (mM) aqueous solutions of SNP/PEH were added drop wise to the previously prepared aqueous solution of α-CD or β-CD making the ultimate equimolar mixture and were continued to stir for 48 hours at 55–60 °C. The suspensions obtained after cooling the mixture to 5 °C were filtered to obtain white crystalline powder, which were then dried in air and preserved in vacuum desiccators for further use.

## Result and Discussion

### Job plot: Stoichiometry of inter molecular association between guest and host

**Figure 2:**
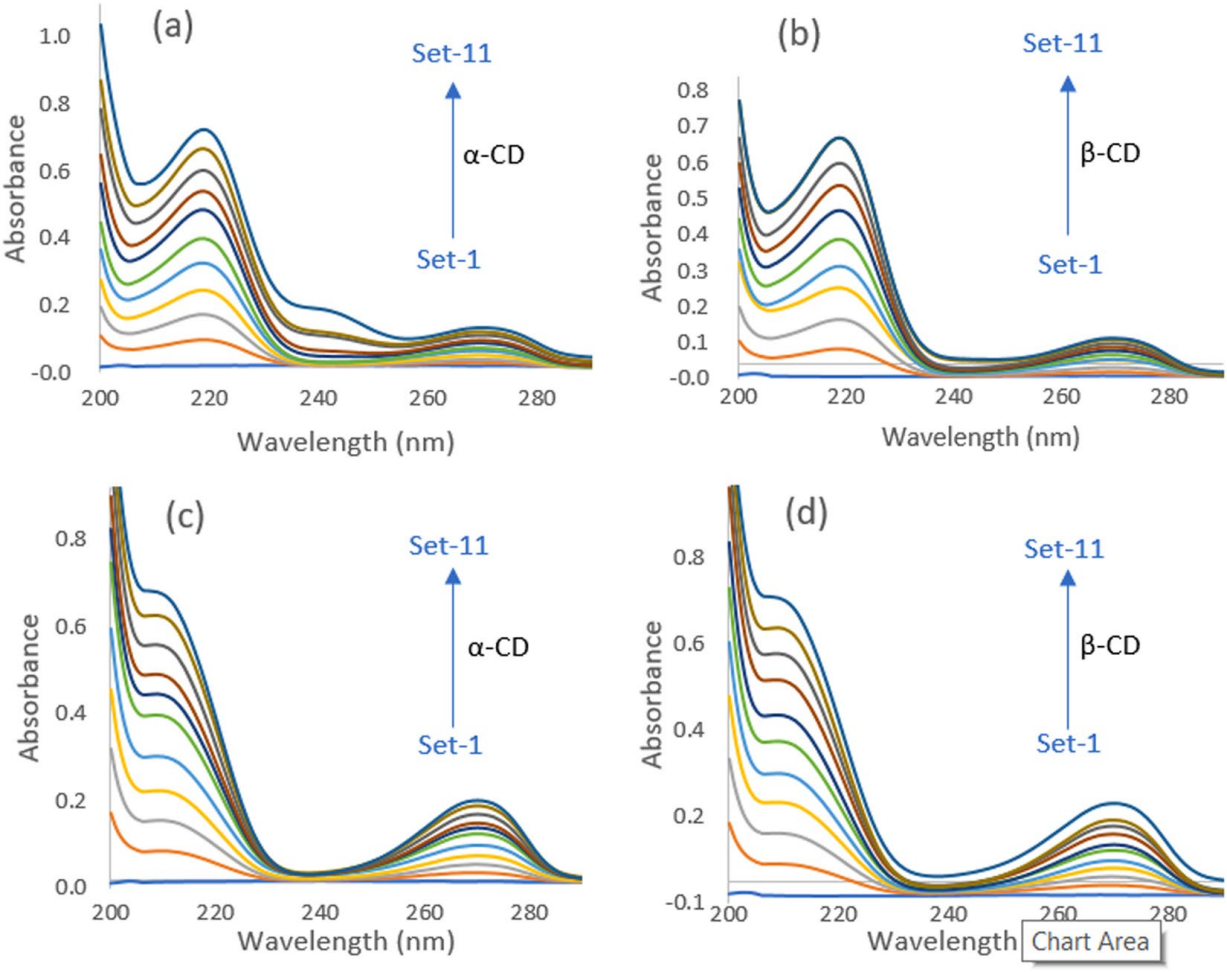
**(a**,**b**,**c**,**d)** UV-Vis spectra for the generation of Job plots of **(a)** SNP + α-CD and **(b)** SNP + β-CD systems at *λ*_max_ = 209 nm, and **(c)** PEH + α-CD and **(d)** PEH + β-CD systems at ${\lambda }_{\max }$ = 219 nm.

**Figure 3:**
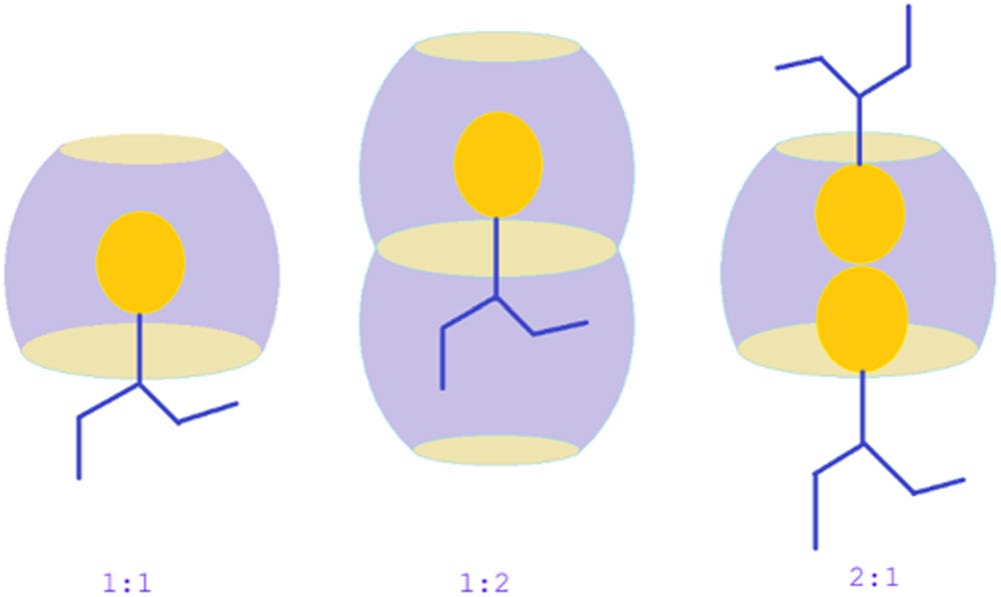
Probable host:guest stoichiometric ratio of the inclusion complexes.

**Figure 4:**
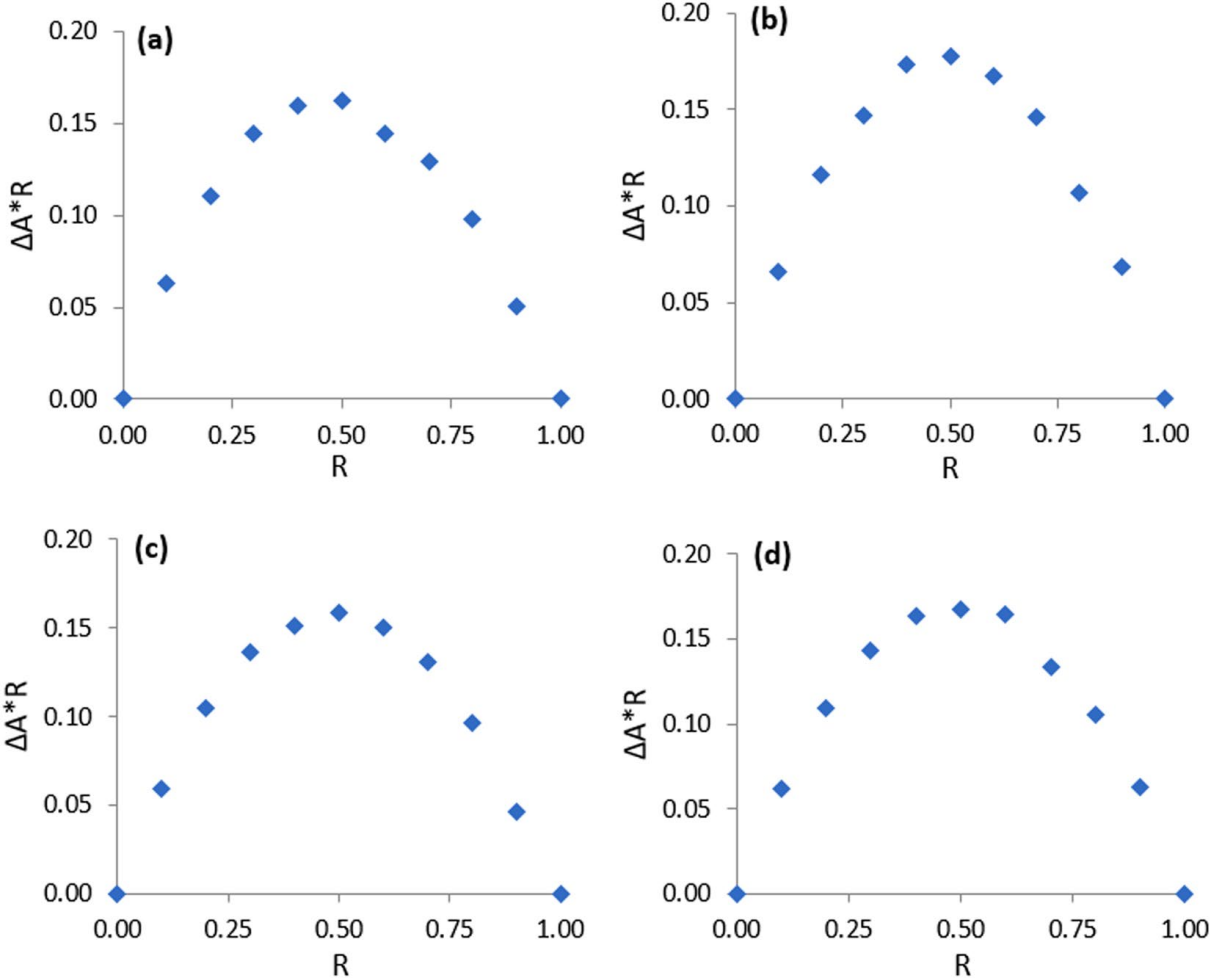
**(a**,**b**,**c**,**d)** Job plots of the **(a)** SNP + α-CD and **(b)** SNP + β-CD systems at ${\lambda }_{\max }$ = 209 nm and **(c)** PEH + α-CD and **(d)** PEH + β-CD systems at ${\lambda }_{\max }$ = 219 nm, at 298.15 K. ΔA = absorbance difference of SNP/PEH without and with CD, R = [DGs]/([DGs] + [CD]).

The stoichiometry of the host-guest inclusion complexes was determined by employing the well-established Jobs method^24^. UV-Vis spectroscopic data were used in this technique to determine the stoichiometry of inclusion complexation. Absorption spectra of a set of solutions, prepared by mixing aqueous SNP/PEH solution with the aqueous α-CD/β-CD solution in the sort of 0–1 mole fraction, were recorded at 298.15 K of temperature. Absorbance of the prepared set of the solutions were taken at λ_max_ = 209 nm for SNP and λ_max_ = 219 nm for PEH (Fig. 2). Jobs plots of (SNP + α-CD, SNP + β-CD, PEH + α-CD, PEH + β-CD) were obtained by plotting a graph, ΔA × R vs R. Where, ΔA is the deference in absorbance between the pure SNP/PEH and the solutions of the set, prepared with CDs (Tables S1–S4 and Fig. 2). R signifies [PEH]/([PEH] + [CD]) and [SNP]/([SNP] + [CD]). The corresponding fractional value of R at maxima of the Jobs plot indicates the stoichiometry of the inclusion complex formed and it is well known that, R = 0.33, 0.5, 0.66 and so on, evidently recommends 1:2, 1:1 and 2:1, Guest:Host stoichiometry of the inclusion complex respectively^25^ (Fig. 3). Ulatowski *et al*. and Hibbert *et al*. showed that Job plot may be used in case of 1:1 complexes, but for other stoichiometries various mathematical models are widely employed^26,27^. In the experimental analysis of the present work, it is found that, for all the four systems (SNP + α-CD) and (SNP + β-CD), (PEH + α-CD), (PEH + β-CD) the value of R = 0.5, clearly indicating the 1:1, Guest:Host stoichiometry of the ICs^28^ (Fig. 4).

### Surface tension: An idea to the Host-Guest molecular association and their stoichiometry in the inclusion complex

**Figure 5:**
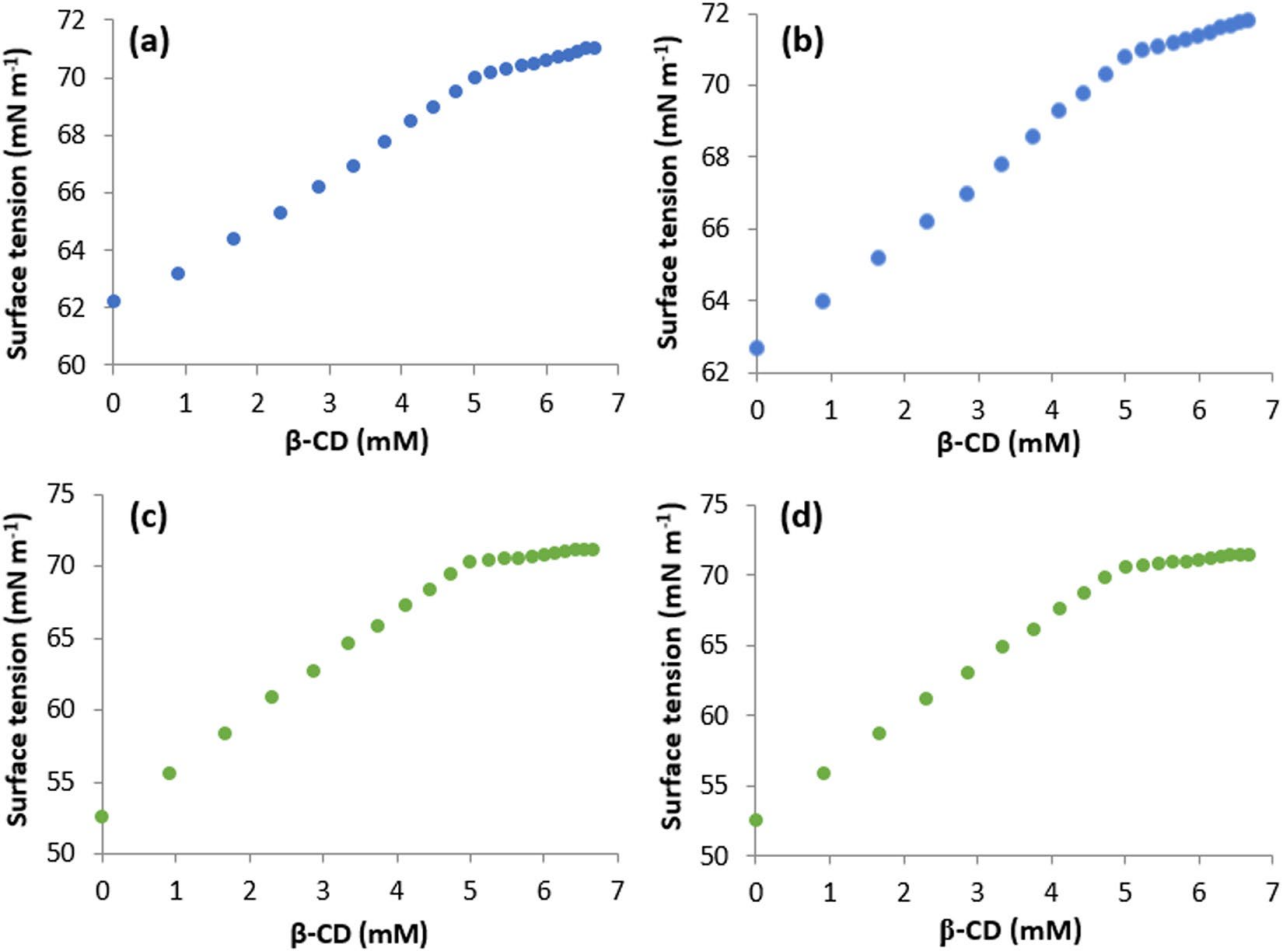
**(a**,**b**,**c**,**d)** Variations in the surface tension of aqueous SNP with increasing concentration of **(a)** α-CD, **(b)** β-CD and the variations in the same of aqueous PEH with increasing concentration of **(c)** α-CD, **(d)** β-CD at 298.15 K.

**Figure 6:**
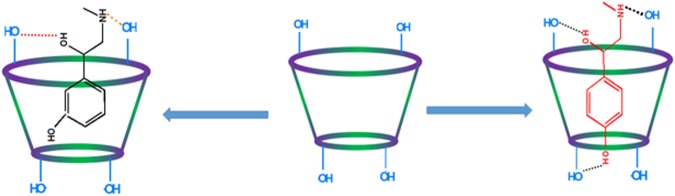
Schematic representation of the host:guest inclusion complexation through the more favorable wider rim of the cyclodextrin molecules.

Sufficiently lower surface tension(γ) value of the aqueous solutions of SNP/PEH than the triply distilled pure water, suggests SNP and PEH to have surface activity. This may be due to the simultaneous presence of phenyl ring as well as the –CH(OH)CH_2_NHCH_3_ group to the opposite terminals of the same molecule. Study on the surface tension of diverse surface-active guest molecules with cyclodextrins strongly supports the inclusion phenomenon and the stoichiometry of the ICs^29–32^. In this work, surface tension of a fixed quantity of aqueous SNP/PEH solutions was studied at 298.15 K with the step wise addition of CDs solutions in same quantity (Tables S5, S6, Fig. 5). Whereas, according to literature as well as practically it is found that, there is an extremely slight change in the surface tension (γ) of CDs over a wide range of concentration in aqueous medium at 298.15 K^33,34^. It signifies all the changes in the value of surface tension (γ) are associated with the SNP/PEH. Being a surface phenomenon, more the number of surface active molecules in the surface of a solution, decreases more the surface tension of that solution. But permanent migration of surface active molecules from the surface to the bulk of the solution by means of solvation or many other stabilizing factors leads to the increase in surface tension (γ) of that solution. This is exactly the trend, what we observed in our experiment during step wise addition of CDs in the aqueous SNP/PEH solution (Tables S5, S6, Fig. 5). This is obviously; there is migration of surface active SNP/PEH molecules from the surface to the bulk of the solution by means of encapsulation of the SNP/PEH into the hydrophobic cavity of the CDs forming host-guest ICs^30,31,35^ (Fig. 6). After a certain concentration of CDs, the surface tension (γ) becomes steady and consequently lefts a sharp break point behind it, in the plot of surface tension (γ) vs concentration of CDs (Table 1, Fig. 5). Accordingly, surface chemistry confers the inclusion phenomenon and appearance of sharp, single break point at the 1:1 molar concentration ratio of host and guest molecules for all the cases (SNP + α-CD, SNP + β-CD, PEH + α-CD, PEH + β-CD) establishes the 1:1 stoichiometry of host-guest ICs^36–39^.

**Table 1:** Values of Surface Tension (γ^a^) at the Break Point with Corresponding Concentrations of DGs and CDs at 298.15 K^a^.

Guest	Host	Concentration of host (mM)	Concentration of guest (mM)	Surface tension (γ^a^) mNm^−1^
PEH	α-CD	4.9379	5.0621	70.1829
	β-CD	4.9276	5.0724	70.4886
SNP	α-CD	5.2152	4.7848	70.1487
	β-CD	5.1564	4.8436	70.4149

^a^Standard uncertainties (u): temperature u(T) = ± 0.01 K, surface tension: u(γ) = ± 0.1 mNm^−1^.

### Conductance: Molecular recognition of guest into host molecules and their stoichiometric ratio in ICs

**Figure 7:**
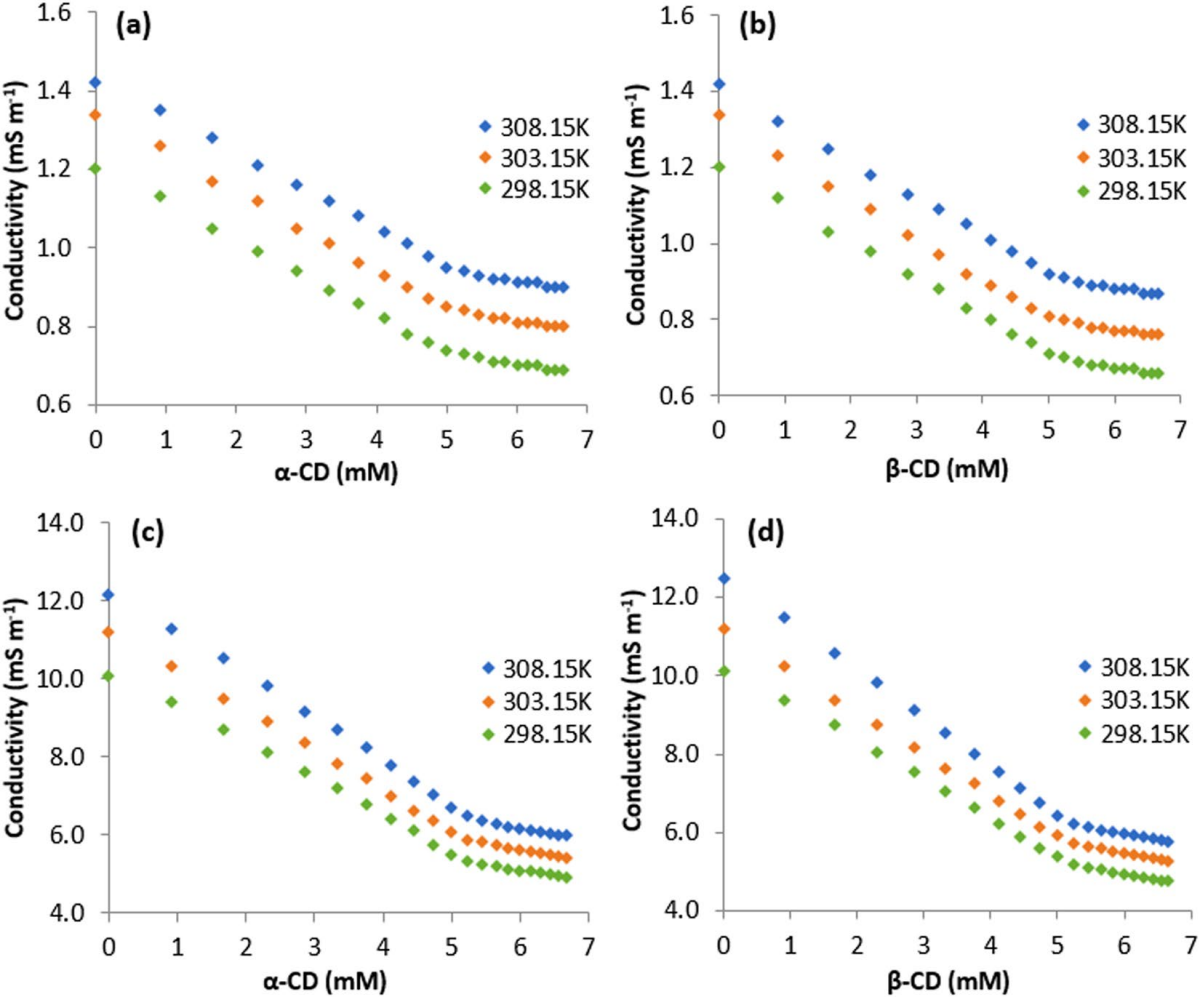
**(a**,**b**,**c**,**d)** Variations in the conductivity of aqueous SNP with increasing concentration of **(a)** α-CD, **(b)** β-CD and the variations in the same of aqueous PEH with increasing concentration of **(c)** α-CD, **(d)** β-CD at 298.15 to 308.15 K.

Conductimetric study is also another approach, which makes us able to conclude about the supramolecular host-guest interaction between the SNP/PEH and CDs and their stoichiometric ratio in the ICs^37,40^. Though, both the SNP and PEH are organic molecules, 10 (mM) aqueous solution of SNP and PEH shows appreciable conductivity. Being a hydrochloride salt, PEH shows higher conductivity than SNP for the same concentration. In the present work, conductivity of SNP and PEH were measured with the step wise increasing concentration of CDs, at three different temperatures from 298.15 K to 308.15 K with the interval of 5 K of temperature (Tables S5, S6 and Fig. 7). It was found that, gradual increase in concentration of CDs leads to the decrease in conductivity (κ), of the aqueous SNP/PEH solutions (Fig. 7). The fruit full explanation for this observation comes through the decrease in the mobility of the conducting species in the solution due to molecular encapsulation of SNP/PEH into the hydrophobic cavity of the CDs^36,41^ (Fig. 6). Generation of a single break point in the conductivity curves after reaching a certain concentration of CDs, suggests, the molecular encapsulation of SNP/PEH into the cavity of CDs is 1:1^31,41^ (Fig. 7). Corresponding concentration of SNP/PEH and CDs at the break points of the conductivity curve are listed in (Table 2). The near about equimolar concentration of SNP/PEH and CDs at the break points of the conductivity curve suggests the 1:1 stoichiometric ratio of the SNP/PEH into CDs of the ICs^36,37^ (Fig. 3).

**Table 2:** Values of Conductivity (κ) at the Break Point with Corresponding Concentrations of DGs and CDs at 298.15 K^a^ to 308.15 K^a^.

Guest	Host	Temperature (K^a^)	Concentration of host (mM)	Concentration of guest (mM)	Conductivity (*K*^a^) (mSm^−1^)
PEH	α-CD	298.15	5.34	4.65	5.29
		303.15	5.21	4.78	5.88
		308.15	4.54	5.45	6.78
	β-CD	298.15	5.21	4.78	5.17
		303.15	5.12	4.87	5.74
		308.15	5.21	4.78	6.20
SNP	α-CD	298.15	5.04	4.95	0.73
		303.15	5.04	4.95	0.84
		308.15	5.23	4.76	0.93
	β-CD	298.15	5.11	4.88	0.70
		303.15	4.94	5.05	0.80
		308.15	5.12	4.87	0.91

^a^Standard uncertainties (u): temperature u(T) = ±0.01 K, conductivity: u(*κ*) = ±0.01 mSm^−1^.

### Ultraviolet Spectroscopy: The association constants (K_a_) and Stability of the of the ICs

The binding ability of the guest into the host molecule and the stability of the inclusion complexes formed were explored by measuring the association constants (K_a_) of the ICs. The UV-vis spectroscopic study enables us to determine the association constant (K_a_) of the ICs in the solution phase^31^. Molar extinction coefficient (Δε) of SNP/PEH, depending upon the solvent polarity, should change while going from polar aqueous media to the apolar hydrophobic cavity of the CDs to form ICs^41,42^. To determine association constant (K_a_), the changes in absorbances (ΔA) of SNP/PEH were measured with increasing concentration of CDs at the temperature range 298.15 K to 308.15 K. (Tables S7–S10) The λ_max_ = 209 nm for SNP and λ_max_ = 219 nm for PEH were considered to determine the association constant (K_a_) in this case (Table 3). According to the Benesi-Hildebrand method to determine the association constant for the 1:1 host-guest inclusion complex, the double reciprocal plot was obtained by using the following equation^31,43–45^.

1 $$\frac{1}{{\rm{\Delta }}A}=\frac{1}{{\rm{\Delta }}\varepsilon [DGs]{K}_{a}}\frac{1}{[CD]}+\frac{1}{{\rm{\Delta }}\varepsilon [DGs]}$$

where, ΔA represents the difference in absorbances of PEH or SNP without CDs to the absorbances of the same with the CDs. [DGs] refers to the concentration of the PEH and SNP. The association constants(${K}_{a}$) of the inclusion complexes, listed in the (Table 3) were obtained by dividing the slope by the intercept of the plot given in the Figs S1, S2.

**Table 3:** Association Constant obtained from Benesi-Hildebrand method (*K*_*a*_), Association Constant obtained from the Nonlinear Program $({K}_{a}^{\theta })$, Association Constant obtained from Program based mathematical calculation of non-linear changes in the conductivity data $({K}_{a}^{C})$, Association Constant obtained from Benesi-Hildebrand equation, using the spectrofluorometric data $({K}_{a}^{F})$ at 298.15 to 308.15 K^a^.

Guest	Host	Temperature (K^a^)	$({{\boldsymbol{K}}}_{{\boldsymbol{a}}})$(×10^−3^)	$({{\boldsymbol{K}}}_{{\boldsymbol{a}}}^{{\boldsymbol{\theta }}})$(×10^−3^)	$({{\boldsymbol{K}}}_{{\boldsymbol{a}}}^{{\boldsymbol{C}}})$(×10^−3^)	$({{\boldsymbol{K}}}_{{\boldsymbol{a}}}^{{\boldsymbol{F}}})$(×10^−3^)
PEH	α-CD	303.15	2.14	2.07	2.05	2.21
		308.15	1.79	1.74	1.72	
		313.15	1.48	1.40	1.38	
	β-CD	303.15	2.97	2.71	2.75	2.91
		308.15	2.26	2.10	2.12	
		313.15	1.79	1.70	1.68	
SNP	α-CD	303.15	2.84	2.82	2.80	2.87
		308.15	2.26	2.15	2.18	
		313.15	1.74	1.63	1.66	
	β-CD	303.15	3.82	3.41	3.35	3.73
		308.15	2.88	2.46	2.42	
		313.15	2.28	1.85	1.85	

^a^Standard uncertainty in temperature, u, are u(T) = ±0.01 K^a^.

UV-vis spectroscopic data were also used in a nonlinear program that practices the changes in absorbance of SNP/PEH due to its molecular recognition into the apolar cavities of CDs and the association constants (${{\rm{K}}}_{{\rm{a}}}^{{\rm{\theta }}}$) were obtained^31,46^. There should be an equilibrium between host and the guest molecules to the formation of 1:1 ICs^47,48^.

2 $${[DGs]}_{f}+{[CD]}_{f}\mathop{\leftrightharpoons }\limits^{{{\rm{K}}}_{a}^{{\rm{\theta }}}}[IC]$$

The expression for the association constant $({K}_{a}^{\theta })$ can be obtained from the above equation as follows-

3 $${K}_{a}^{\theta }=\frac{[IC]}{{[DGs]}_{f}{[CD]}_{f}}$$

where, [IC], [*DGs*]_*f*_ and [*CD*]_*f*_ represents the concentration of inclusion complex, free SNP/PEHand cyclodextrin respectively at the equilibrium of the reaction. The equation for the association constant (${{\rm{K}}}_{{\rm{a}}}^{{\rm{\theta }}}$) can also be expressed as the absorbances of the host and the guest molecules as follows-

4 $${K}_{a}^{\theta }=\frac{[IC]}{{[DGs]}_{f}{[CD]}_{f}}=\frac{({A}_{obs}-{A}_{0})}{(A-{A}_{obs}){[CD]}_{f}}$$

Here,

5 $${[CD]}_{f}={[CD]}_{x}-\frac{{[DGs]}_{x}({A}_{obs}-{A}_{0})}{(A-{A}_{obs})}$$

where, A_0_ is the absorbance of SNP/PEH molecules in the initial state, A_obs_ denotes the absorbances of the same during the gradual addition of CDs and A refers to the final concentration of SNP/PEH molecules. [CD]_x_ and [DGs]_x_ is the concentration of cyclodextrins added and SNP/PEH molecules respectively. The association constants (${{\rm{K}}}_{{\rm{a}}}^{{\rm{\theta }}}$), obtained from the binding isotherm with the application non-linear program are listed in the Table 3.

### Conductance: Program based mathematical calculation of non-linear changes in the conductivity data and association constants $({K}_{a}^{C})$

Non-linear changes in the conductivity data at the temperature ranging from 298.15 K to 308.15 K were utilized in the mathematical program and the association constants ${(K}_{{\rm{a}}}^{{\rm{c}}})$ for1:1 DGs-CDs ICs, listed in the Table 3 are frequently obtained^49–51^. The complexation reaction between DGs and CDs to produce ICs is supposed to proceed via the following chemical equilibrium

6 $${[DGs]}_{f}+{[CD]}_{f}\mathop{\leftrightharpoons }\limits^{{{\rm{K}}}_{a}^{C}}\,\,[IC]$$

The above equation can be reduced to the following form to find out the association constant ${K}_{a}^{c}$

7 $${K}_{a}^{c}=\frac{[IC]}{{[DGs]}_{f}{[CD]}_{f}}$$

Here, $[IC]$ is the equilibrium concentration of inclusion complexes, ${[DGs]}_{f}$ and ${[CD]}_{f}$ refers to the concentration of SNP/PEH and CDs in the free state.

The association constant $({K}_{a}^{c})$ can be calculated in terms of conductivities from the various non-linear isotherm as follows^50,51^-

8 $${K}_{a}^{c}=\frac{[IC]}{{[DGs]}_{f}{[CD]}_{f}}=\frac{({\kappa }_{obs}-{\kappa }_{0})}{(\kappa -{\kappa }_{obs}){[CD]}_{f}}$$

Where,

9 $${[CD]}_{f}={[CD]}_{ad}-\frac{{[DGs]}_{ad}({\kappa }_{obs}-{\kappa }_{0})}{(\kappa -{\kappa }_{0})}$$

Here, ${\kappa }_{0},{\kappa }_{obs}$ and $\kappa$ corresponds to the conductivities of DGs at initial state, during addition of CDs and the final state respectively. Instantaneous concentration of DGs while addition of CDs is represented by ${[DGs]}_{ad}$ and ${[CD]}_{ad}$ is the concentration of the added CDs.

### Fluorescence: Modified Benesi-Hildebrand equation and association constants

The association constants $({{\rm{K}}}_{{\rm{a}}}^{{\rm{F}}})$ of the ICs in the solution phase were also determined using the spectrofluorometric data and the association constants, determined are found in good agreement with the data obtained from all the previously described methodology^52–54^ (Table 3, Tables S11–S14). An enhancement of the intensities of the spectral lines accompanied by the slight hypsochromic shift were observed with the step wise increase in concentration of the CDs (α and β-cyclodextrins) solutions (Figs S3, S4). The observations enriched us with the knowledge that, a change in the molar extinction coefficient i.e. polarity of the environment, surrounding the chromophore, leaded by the encapsulation of the chromophore of the guest molecules (SNP and PEH) from the polar aqueous environment to the apolar hydrophobic cavity of the cyclodextrins. Sometimes, the enhancement in the intensities of the spectral lines are experienced due to the shielding of the excited singlet species of the chromophores from quenching and non-radiative decay with the protective microenvironment created by the hollow-circular, apolar cavity of the CDs^55,56^. The spectrofluorometric data were analyzed and run with the modified Benesi-Hildebrand equation to generate the double reciprocal plots, (Figs S5, S6) and the association constants (${{\rm{K}}}_{{\rm{a}}}^{{\rm{F}}}$) of the ICs were obtained as the ratio of the intercept to slope of the plots.

10 $$\frac{1}{I-{I}_{0}}=\frac{1}{[{I}^{/}-{I}_{0}]{K}_{a}^{F}}\frac{1}{[CD]}+\frac{1}{{I}^{/}-{I}_{0}}$$

where, $I$ and ${I}_{0}$ represents the fluorescence intensities of SNP/PEH in the presence and absence of the CDs respectively, ${I}^{/}$ are the intensities of the SNP/PEH while all the guest molecules for a particular system are complexed with CDs. [CD] represents the concentration of the cyclodextrins (α and β-CD).

### UV-vis spectroscopy, Non-linear program based mathematical calculation, Fluorescence and Conductance: Association constants and the thermodynamic parameters

According to the Van’t Hoff Eq. (11) the various thermodynamic parameters for the formation of the inclusion complexes were derived from the ready available association constants (${K}_{a}$, ${{\rm{K}}}_{{\rm{a}}}^{{\rm{\theta }}}$, ${K}_{a}^{C}$) obtained from Benesi-Hildebrand equation, nonlinear methods and non-linear changes in the conductivity data^31,37,41^. (Tables 4, S15–S20, Figs S7–S12)

11 $$\mathrm{ln}\,{K}_{a}=-\,\frac{{\rm{\Delta }}{H}^{0}}{RT}+\frac{{\rm{\Delta }}{S}^{0}}{R}$$

**Table 4:** Thermodynamic parameters (ΔH°, ΔS°, ΔG°) calculated, using the association constants (${K}_{a}$, ${K}_{a}^{\theta }$, ${K}_{a}^{C}$) obtained from Benesi-Hildebrand method, nonlinear Program, program based mathematical calculation of non-linear changes in the conductivity data respectively.

Inclusion Complexes (ICs)	Application of (${{\boldsymbol{K}}}_{{\boldsymbol{a}}}$) to Van’t Hoff equation		Application of (${{\boldsymbol{K}}}_{{\boldsymbol{a}}}^{{\boldsymbol{\theta }}}$) to Van’t Hoff equation		Application of (${{\boldsymbol{K}}}_{{\boldsymbol{a}}}^{{\boldsymbol{C}}}$) to Van’t Hoff equation	
PEH + α-CD	ΔH° (KJ mol^−1^)	−28.93	ΔH^0θ^ (KJ mol^−1^)	−30.85	ΔH^0C^ (KJ mol^−1^)	−31.22
	ΔS° (J mol^−1^ K^−1^)	−31.69	ΔS^0θ^ (J mol^−1^ K^−1^)	−38.20	ΔS^0C^ (J mol^−1^ K^−1^)	−39.50
	ΔG° (KJ mol^−1^)	−19.49	ΔG^0θ^ (KJ mol^−1^)	−19.46	ΔG^0C^ (KJ mol^−1^)	−19.44
PEH + β-CD	ΔH^0^ (KJ mol^−1^)	−40.15	ΔH^0θ^ (KJ mol^−1^)	−36.82	ΔH^0C^ (KJ mol^−1^)	−38.91
	ΔS^0^ (J mol^−1^ K^−1^)	−66.01	ΔS^0θ^ (J mol^−1^ K^−1^)	−55.79	ΔS^0C^ (J mol^−1^ K^−1^)	−62.52
	ΔG^0^ (KJ mol^−1^)	−20.47	ΔG^0θ^ (KJ mol^−1^)	−20.19	ΔG^0C^ (KJ mol^−1^)	−20.27
SNP + α-CD	ΔH^0^ (KJ mol^−1^)	−38.72	ΔH^0θ^ (KJ mol^−1^)	−43.26	ΔH^0C^ (KJ mol^−1^)	−41.25
	ΔS^0^ (J mol^−1^ K^−1^)	−61.54	ΔS^0θ^ (J mol^−1^ K^−1^)	−76.63	ΔS^0C^ (J mol^−1^ K^−1^)	−70.04
	ΔG^0^ (KJ mol^−1^)	−20.37	ΔG^0θ^ (KJ mol^−1^)	−20.41	ΔG^0C^ (KJ mol^−1^)	−20.37
SNP + β-CD	ΔH^0^ (KJ mol^−1^)	−40.81	ΔH^0θ^ (KJ mol^−1^)	−48.37	ΔH^0C^ (KJ mol^−1^)	−46.89
	ΔS^0^ (J mol^−1^ K^−1^)	−66.11	ΔS^0θ^ (J mol^−1^ K^−1^)	−91.96	ΔS^0C^ (J mol^−1^ K^−1^)	−87.24
	ΔG^0^ (KJ mol^−1^)	−21.10	ΔG^0θ^ (KJ mol^−1^)	−20.95	ΔG^0C^ (KJ mol^−1^)	−20.88

Mean errors in variables are as follows: ΔH^0^ = ±0.01 kJ mol^−1^; ΔS^0^ = ±0.01 J mol^−1^K^−1^; ΔG^0^ = ±0.01 kJ mol^−1^; ΔH^0θ^ = ±0.01 kJ mol^−1^; ΔS^0θ^ = ±0.01 J mol^−1^K^−1^; ΔG^0θ^ = ±0.01 kJ mol^−1^; ΔH^0C^ = ±0.01 kJ mol^−1^; ΔS^0C^ = ±0.01 J mol^−1^K^−1^; ΔG^0C^ = ±0.01 kJ mol^−1^.

Calculation on the thermodynamic parameters of the formation of ICs, it is found that, both the changes in entropy and enthalpy of formation appears to be negative, suggesting an exothermic and entropy restricted rather than entropy driven process (Table 4). The explanation on the decrease in entropy during inclusion complexation comes from the molecular association of the host and guest molecules to form inclusion complexes in the solution. Though the process is entropy restricted, the negative value of the enthalpy (${\rm{\Delta }}{H}^{0},{\rm{\Delta }}{H}^{\theta 0}$, ${\rm{\Delta }}{H}^{C0}$) makes the overall energy negative i.e. negative ${{\rm{\Delta }}G}^{{\rm{0}}}$ and finds its spontaneity in the formation of ICs (Table 4).

### ^1^H NMR and 2D ROESY NMR spectra analysis

Inclusion of a molecule inside into the cavity of cyclodextrin consequences in the chemical shift of the interacting protons of both the guest and cyclodextrin in ^1^H NMR spectra, due to their mutual shielding through space^57^. Encapsulation of aromatic guest molecule results the diamagnetic shielding of the interacting protons of cyclodextrin by the aromatic moiety^58^ (Table S21). Cyclodextrin molecule has H3 and H5 hydrogens at inside of the conical cavity, specially, the H3 are located near the wider rim while H5 are positioned near the narrower rim and the other H1, H2 and H4 hydrogens are situated at the exterior of the cyclodextrin molecule (Fig. 1)^59^. In this work the molecular inclusions have been studied with the help of ^1^H NMR spectra. The ^1^H NMR spectra of the pure α-CD, β-CD, SNP + α-CD, SNP + β-CD, PEH + *α*-CD and PEH + β-CD systems are shown in Figs S13–S18 respectively, where the aromatic as well as signals of H3 and H5 protons of cyclodextrins may be observed with corresponding chemical shift (δ) values. The ^1^H NMR spectra of the complexes reveal that the signals of interior H3 and H5 of α and β-CD plus that of the interacting aromatic protons of SNP/PEH showed substantial upfield shift confirming the formation of inclusion complexes^60^ (Figs S13–S18).

**Figure 8:**
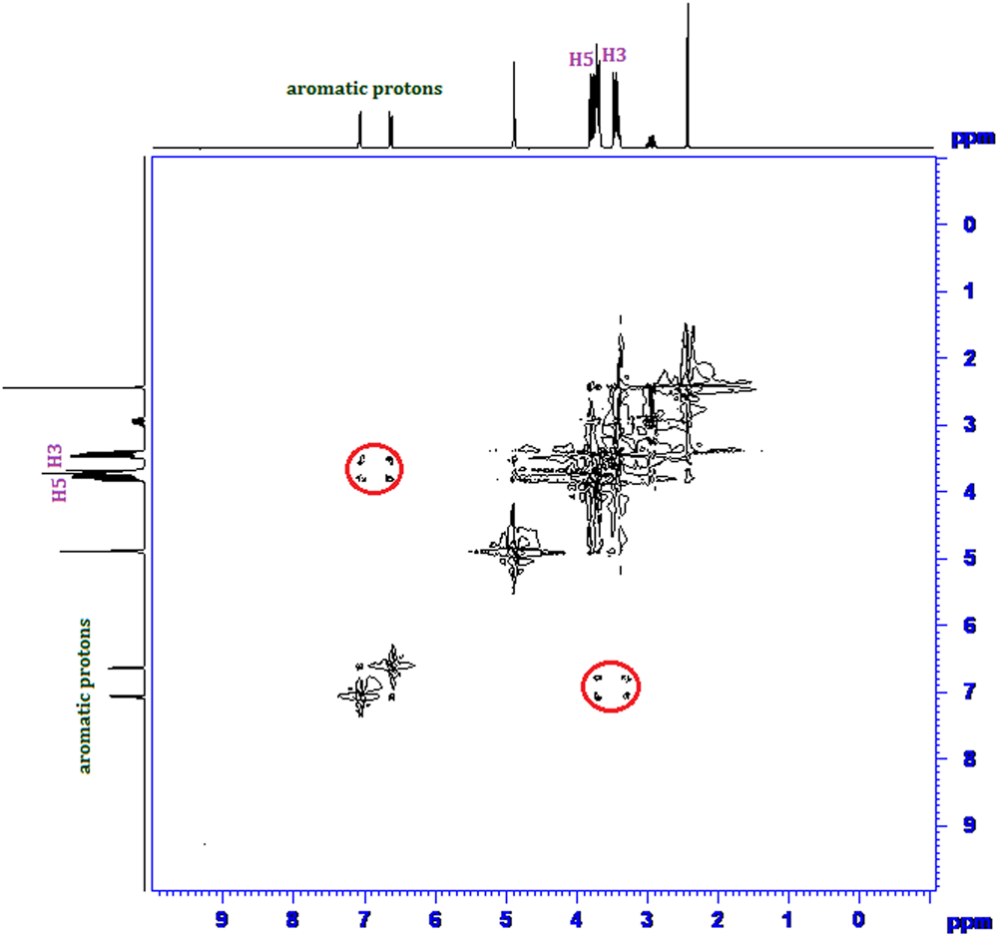
2D ROESY NMR spectra of the solid (SNP + α-CD) system.

**Figure 9:**
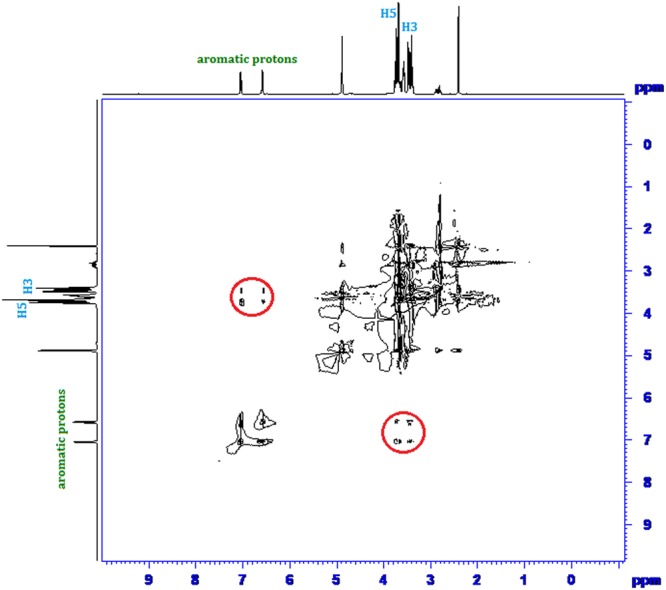
2D ROESY NMR spectra of the solid (SNP + β-CD) system.

**Figure 10:**
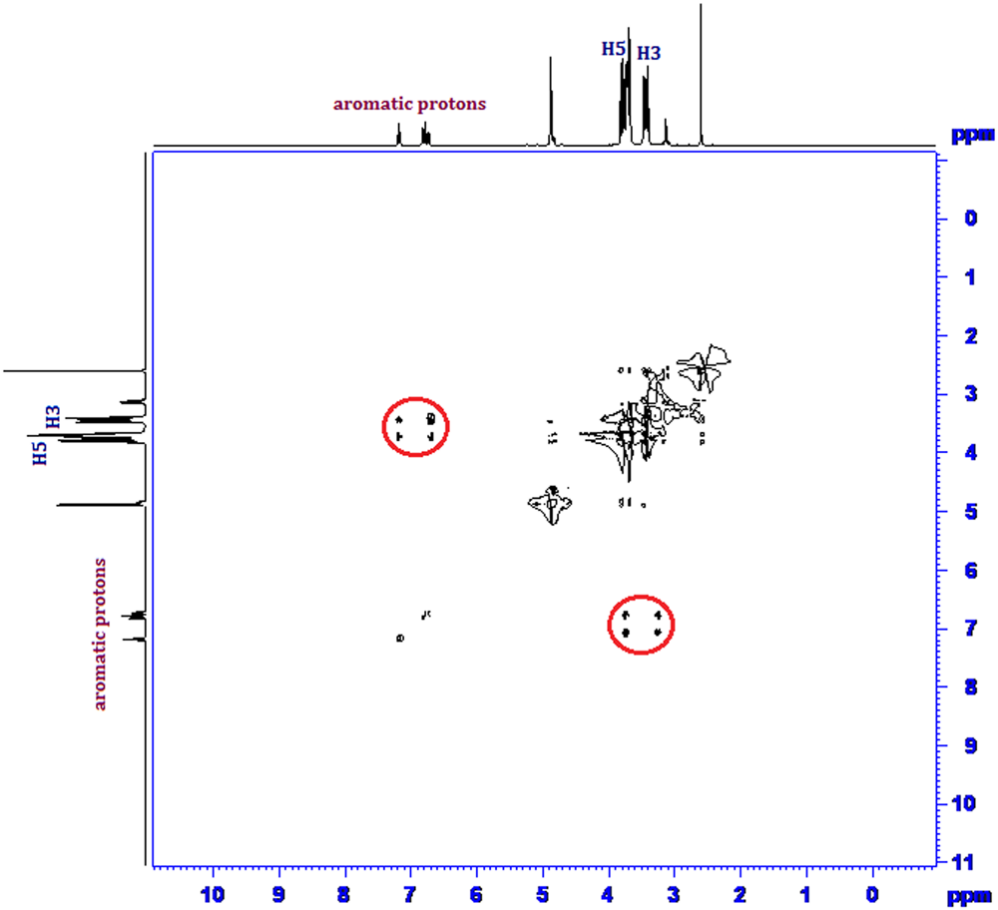
2D ROESY NMR spectra of the solid (PEH + α-CD) system.

**Figure 11:**
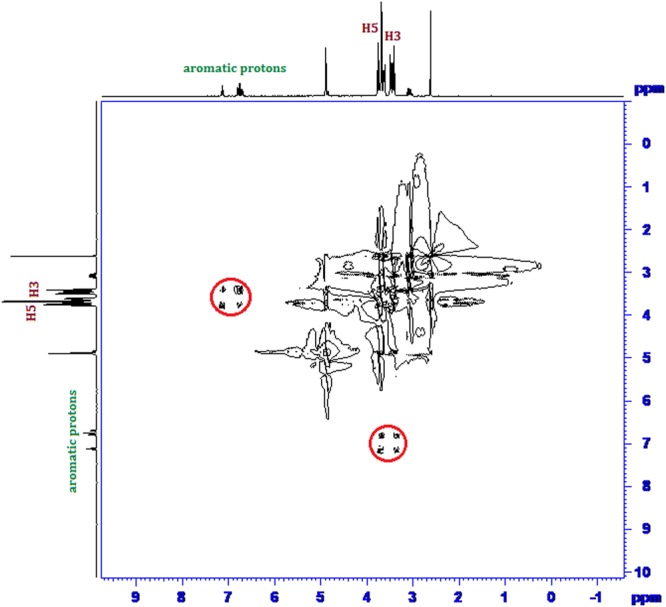
2D ROESY NMR spectra of the solid (PEH + β-CD) system.

2D ROESY NMR spectroscopy provides decisive evidence about the spatial closeness of the interacting atoms of the host and the guest by observing the intermolecular dipolar cross-correlations^61,62^. The protons which are situated within 0.4 nm in space may produce a rotating-frame NOE spectroscopy (ROESY)^63^. According to structure of α and β-CD, inclusion phenomenon inside into cyclodextrin cavity can be shown by the appearance of NOE cross-peaks between the protons of cyclodextrin and the protons of the aromatic guest identifying their spatial proximity^64,65^. To prove this, 2D ROESY spectra of the complexes of SNP and PEH with α and β-CD in D_2_O, were recorded, which shows significant correlation of aromatic protons of SNP and PEH with the H3 and H5 protons of α and β-CD, establishing the aromatic ring was encapsulated inside both the cyclodextrin cavities^66^ (Figs 8–11**)**. It may be detected that the H-6 protons of cyclodextrins were not influenced by the inclusion processes, suggesting that the SNP/PEH molecule was incorporated into the cyclodextrin cavity via the wider rim, not through the narrower rim as otherwise cross-peaks between the H6 and the guest would have been observed in the ROESY spectra^67^ (Fig. 6).

### HRMS Analysis of Inclusion complexes

**Figure 12:**
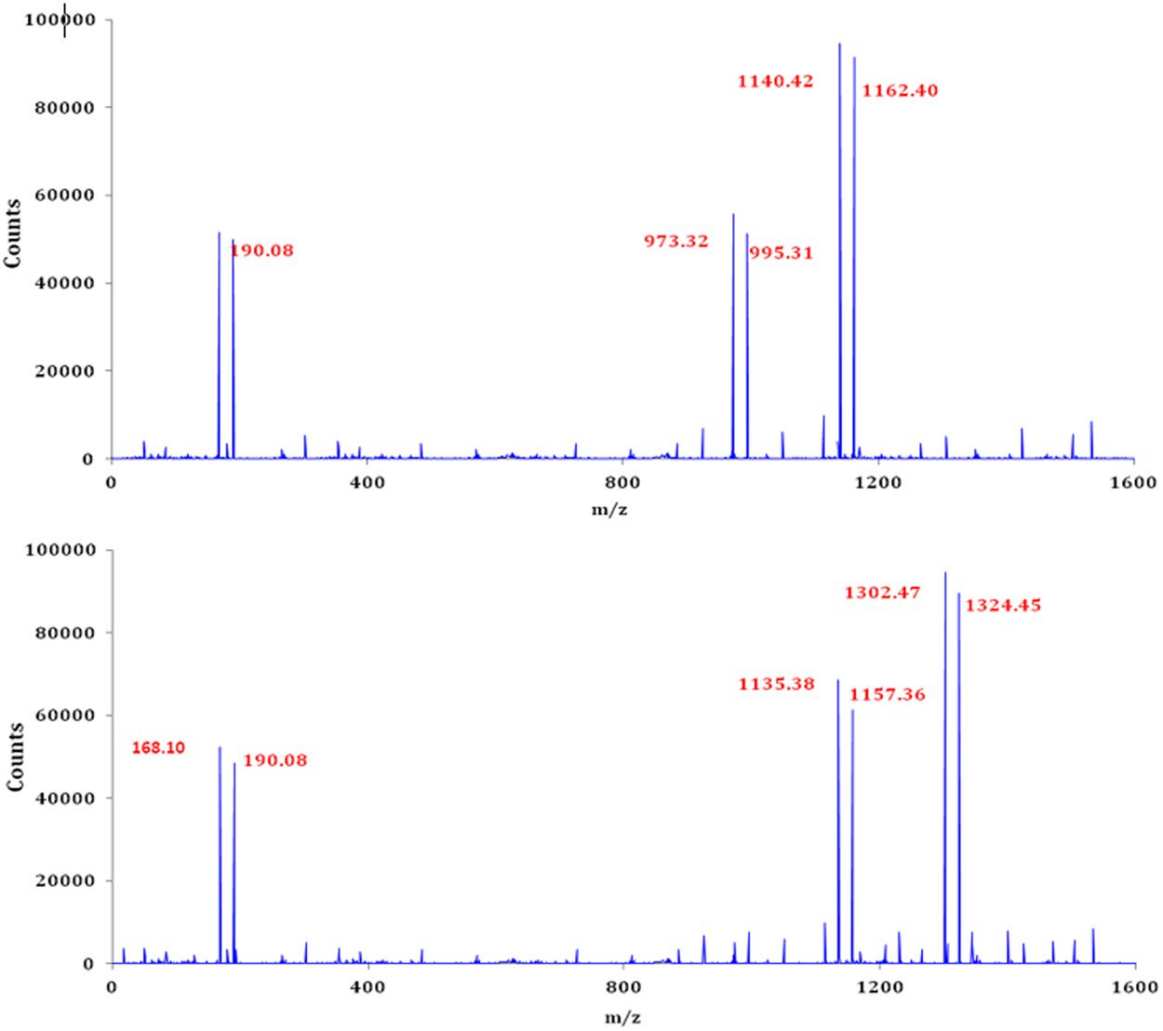
**(a**,**b)** HRMS spectra of the **(a)** SNP + α-CD, PEH + α-CD and **(b)** SNP + β-CD, PEH + β-CD ICs.

Mass spectroscopic study of the solid inclusion complexes of SNP/PEH with α and β-CD were done after the dissolution of the ICs in methanol. The spectra are shown in the Fig. 12 and Table S22. enlists the m/z values for the corresponding fragmentations added to the molecular ion peak. The appearance of peaks at the m/z 1140.42 and 1162.40 corresponds to the [SNP/PEH + α-CD + H]^+^ and [SNP/PEH + α-CD + Na]^+^ respectively and the peaks at 1302.47 and 1324.45 corresponding to the [SNP + β-CD + H]^+^ and [SNP + β-CD + Na]^+^ respectively. The tangible existence of the peaks in the spectra mentioned above approves the formation of the inclusion complexes i.e. [PEH + α-CD], [PEH + β-CD], [SNP + α-CD] and [SNP + β-CD] and their host – guest stoichiometric ratio should be 1:1 (Fig. 3)^68,69^.

### FTIR spectroscopy

**Figure 13:**
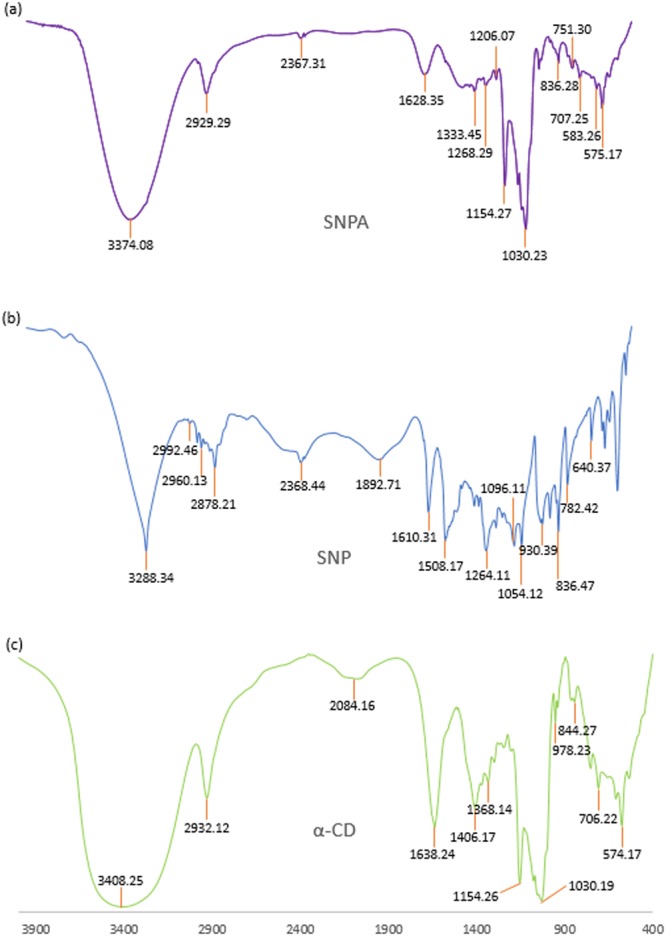
**(a**,**b**,**c)** FTIR spectra of **(a)** SNP + α-CD, **(b)** SNP, **(c)** α-CD.

**Figure 14:**
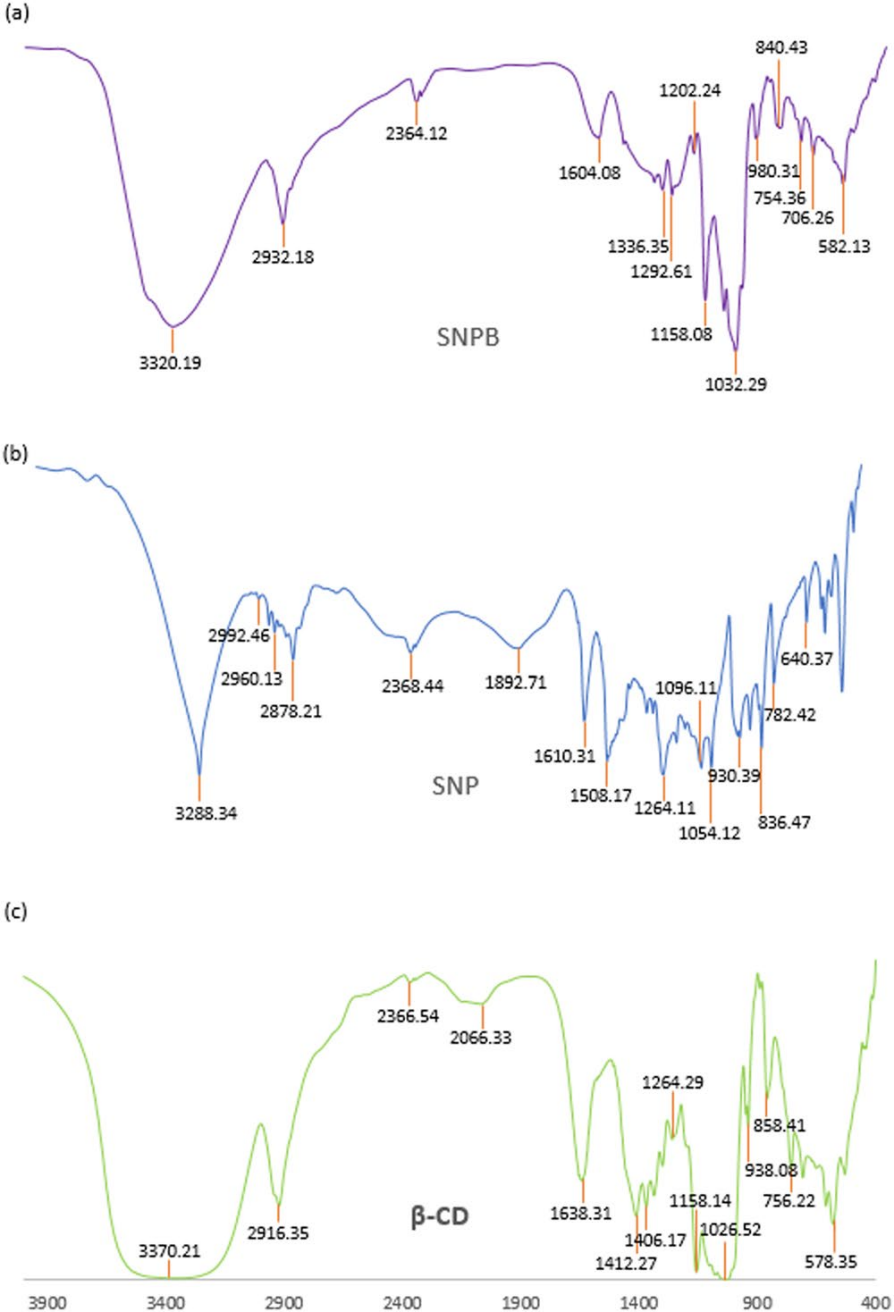
**(a**,**b**,**c)** FTIR spectra of **(a)** SNP + β-CD, **(b)** SNP, **(c)** β-CD.

**Figure 15:**
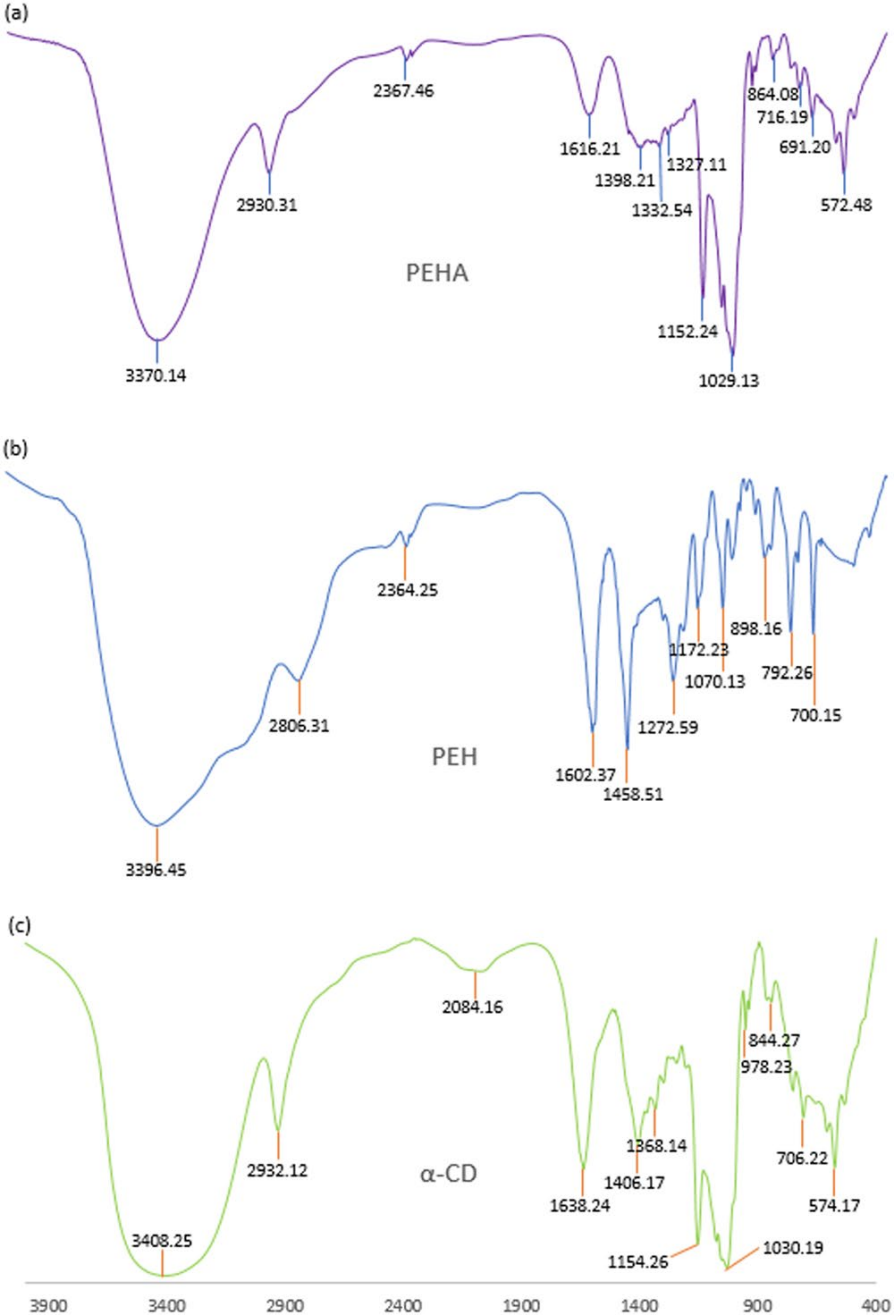
**(a**,**b**,**c)** FTIR spectra of **(a)** PEH + α-CD, **(b)** PEH, **(c)** α-CD.

**Figure 16:**
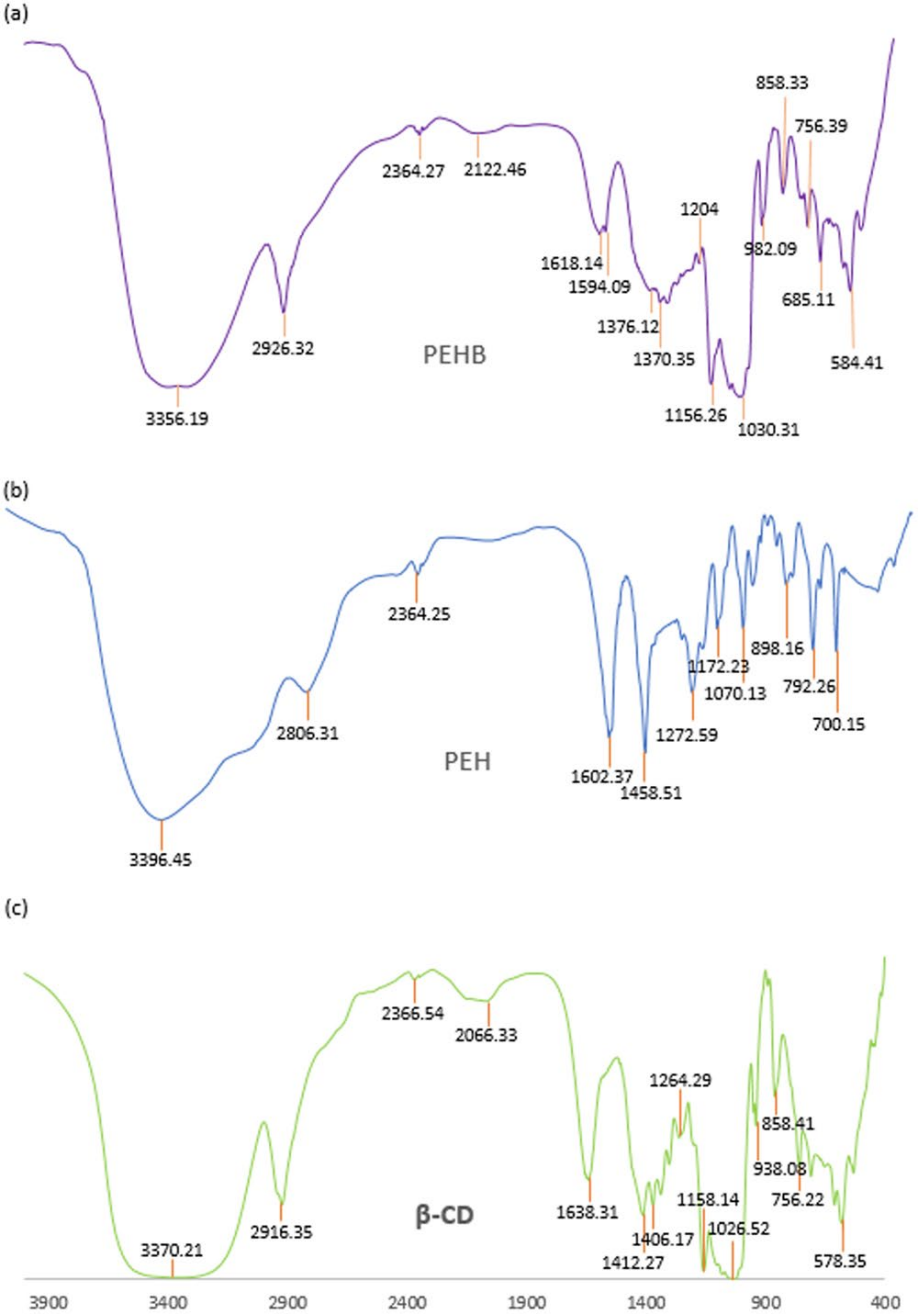
**(a**,**b**,**c)** FTIR spectra of **(a)** PEH + β-CD, **(b)** PEH, **(c)** β-CD.

Interpretation of the Infra-red spectroscopic data of the ICs as well as the pure host and guest molecules also reveals the veracity about the way by which the ICs are formed and supports the same circumstances of host – guest interaction as obtained from the 2D ROESY NMR spectroscopic study^70,71^. All the FTIR spectra of the solid inclusion complexes and the pure host and guest molecules were recorded by preparing KBr disk. The changes in the significant peak values in the IR spectra on going from the pure host and guest molecules to the inclusion complexes which are shown in the Figs 13–16, suggests the formation of ICs exploring the binding mode of the guests to the host molecules^60,72^. The IR stretching frequencies (cm^−1^) of noteworthy responsible for the corresponding chemical bonds are listed in the Table S23.

Analysis of the FTIR spectra for the [SNP + α-CD] along with the spectra of pure α-CD and SNP: (i) The -O-H stretching frequency of the α-CD and the -O-H and -N-H stretching frequencies of SNP were observed at 3408.25 and 2992.46 to 2960.13 cm^−1^ respectively, which appears as a broad peak at 3374.08 cm^−1^ in case of the IC. The responsible fact for this shifting in frequencies is the formation of H-bond between SNP and α-CD. (ii) The peaks at 1054.12 and 1264.11 cm^−1^ responsible for the -C-O stretching for secondary and phenolic -C-OH group of SNP respectively are shifted to 1030.23 and 1154.27 cm^−1^ correspondingly for the [SNP + α-CD] IC. Thus, weakening of -C-O bond proposes the formation of H-bond via the phenolic as well as the secondary -OH group of the SNP molecule. (iii) The stretching and bending frequencies for the -C-H bond of the α-CD was at 2932.12 and 1406.17 cm^−1^ and -C-H the out-of-plane bending frequencies for SNP were at 782.42 and 640.37 cm^−1^. But in case of IC their existence is observed at 2929.30, 1333.02, 707.25 and 583.26 cm^−1^, suggesting the various interactions taking place between SNP and α-CD (Fig. 13).

Innumerable interactions of the SNP and β-CD in the [SNP + β-CD] IC were analyzed as follows- (i) The signal for -O-H stretching of β-CD was at 3370.21 cm^−1^and the -O-H and -N-H stretching frequencies of SNP were at 3288.34 cm^−1^ and the region of 2992.46 to 2960.13 cm^−1^ respectively, whereas in the IC these signals shifted to 3320.14 and 2933.18 cm^−1^ correspondingly. This is possibly due to the formation of H-bonding between SNP and β-CD. (ii) The peaks for -C-O (secondary and phenolic -C-OH group) of SNP were at 1054.12 and 1264.11 cm^−1^, which shifted to 1032.29 and 1158.08 cm^−1^ respectively. This is probably owing to the formation of H-bond between SNP and β-CD. (iii) The signals at 2916.35 cm^−1^ and 1412.27 cm^−1^ corresponding to -C-H stretching and -C-H bending of β-CD, shifted to 2932.18 cm^−1^ and 1336.35 cm^−1^ respectively. On the other hand, -C-H out-of-plane bending for SNP molecule were observed at 782.42 cm^−1^ and 640.37 cm^−1^, which shifted to 754.36 cm^−1^ and 582.13 cm^−1^ correspondingly. This may because of the various interactions taking place while the formation of the supramolecular assembly between SNP and β-CD (Fig. 14).

The various interactions that may cause the following spectroscopic changes in the [PEH + α-CD] IC are: (i)The peak for -O-H of α-CD was at 3408.25 cm^−1^ and the phenolic -O-H and -N-H stretching frequencies of PEH appeared as a broad peak at 3028.17 to 3396.45 cm^−1^. But, in case of IC it is shifted to 3370.14 cm^−1^ indicating the formation of H-bond between PEH and CD. (ii) The -C-O stretching frequencies of PEH was at 1070.06 cm^−1^ (secondary alcohol) and 1272.59 cm^−1^ (phenol) where as these are shifted to 1029.13 cm^−1^and 1152.24 cm^−1^ respectively in case of IC, suggesting the formation of H-bond through the secondary and phenolic H-atom of the PEH molecule. (iii) The signal of the -C-H stretching and -C-H bending mode of the α-CD were at 2932.12 and 1406.17 cm^−1^ respectively and the aromatic out-of-plane -C-H bending of PEH were at 792.26 and 700.15 cm^−1^ respectively, which appeared in case of the IC at the frequencies 2930.31, 1398.21, 716.19 and 690.20 cm^−1^ respectively, shifting of these signals leads to the ready explanation that, the close proximity of the -C-H of the α-CD with the aromatic -C-H of the PEH as obtainable from the 2D ROESY spectra. (Fig. 15).

The shifting of the following IR signals satisfactorily explicates the formation of [PEH + β-CD] IC. (i) The -O-H signal for β-CD was at 3370.21 cm^−1^ and the phenolic -O-H and -N-H were at 3028.17 to 3396.45 cm^−1^ which are shifted to 3356.19 cm^−1^ for IC. This is probably the formation of the H-bond of PEH with β-CD. (ii) The peaks at the 1070.06 cm^−1^ (-C-O, secondary alcohol) and 1272.59 cm^−1^ (-C-O, phenolic) for the PEH were shifted to the frequencies 1030.34 cm^−1^ and 1156.31 cm^−1^, validates the participation of secondary and phenolic -O-H group of PEH towards the formation of H-bond with β-CD. (iii) The -C-H stretching and bending mode of frequencies of β-CD were at 2918.35 and 1412.27 cm^−1^ respectively and peaks for the aromatic out-of-plane -C-H bending frequencies for PEH were at792.26 and 700.15 cm^−1^ respectively, are now shifted to 2926.32, 1376.12,756.39 and 685.11 cm^−1^ respectively. Thus, FTIR spectral analysis also indorses the same as obtained from the 2D ROESY spectra (Fig. 16).

There is no sign of chemical reaction taking place while the formation of all the inclusion complexes, exemplified by the no appearance of additional signal in the IR spectra suggesting, all the shifting in signals appearing are responsible for the formation of ICs.

### Scanning Electron Microscope (SEM)

**Figure 17:**
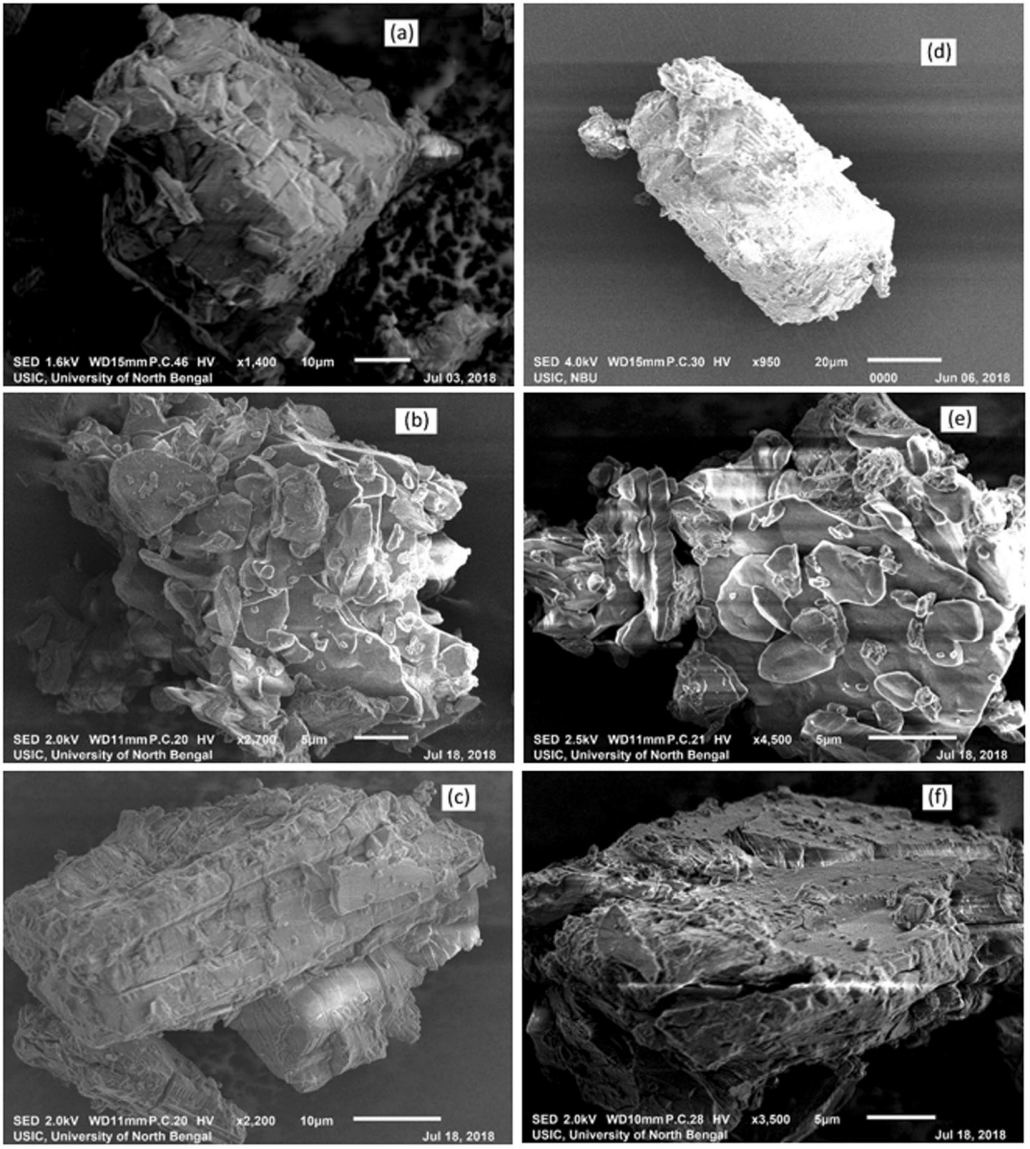
**(a**,**b**,**c**,**d**,**e**,**f)** SEM images of **(a)** α-CD, **(b)** (SNP + α-CD) physical mixture, **(c)** (SNP + α-CD) inclusion complex **(d)** β-CD, **(e)** (SNP + β -CD) physical mixture, **(f)** (SNP + β-CD) inclusion complex.

**Figure 18:**
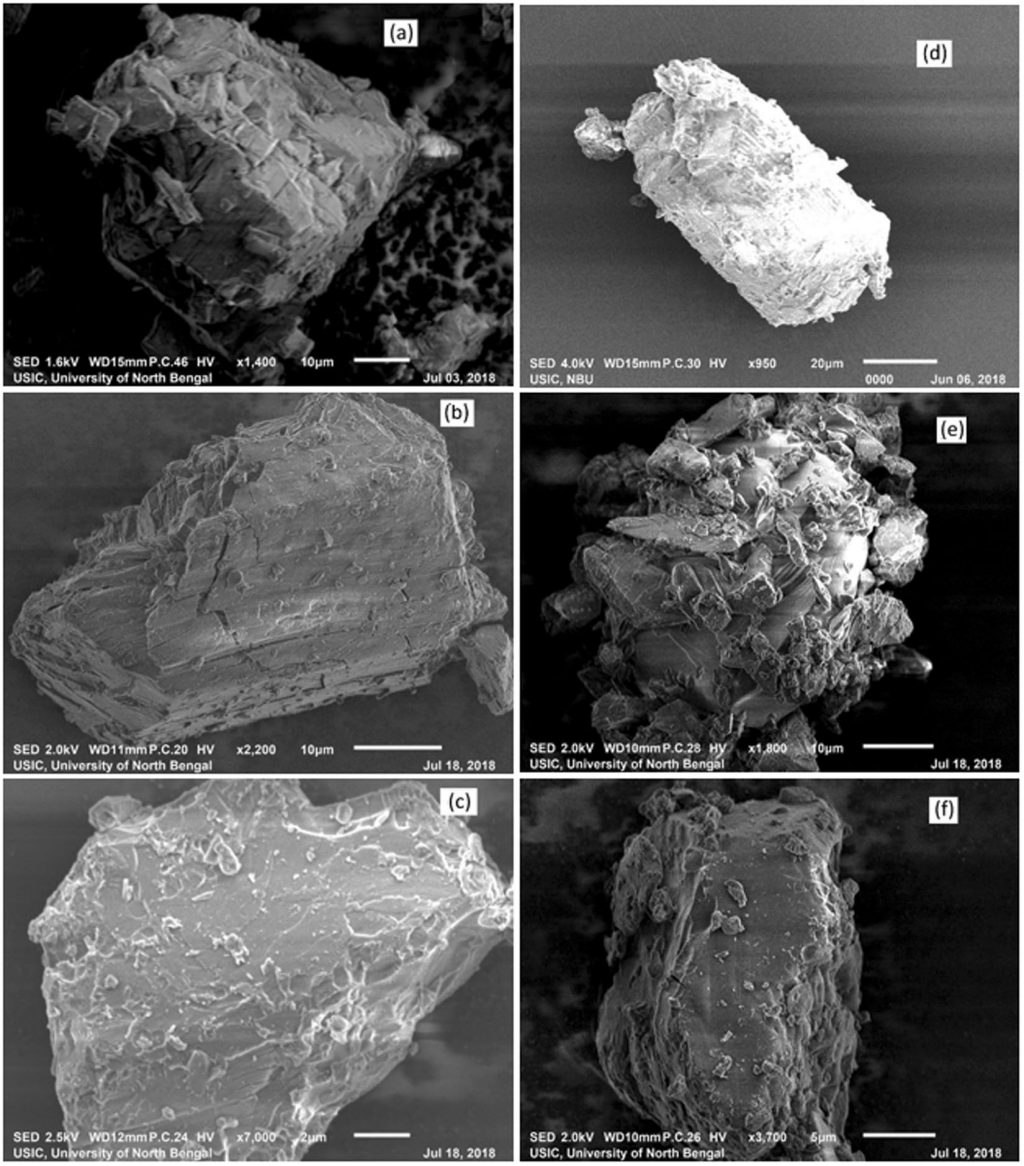
**(a**,**b**,**c**,**d**,**e**,**f)** SEM images of **(a)** α-CD, **(b)** (PEH + α-CD) physical mixture, **(c)** (PEH + α-CD) inclusion complex **(d)** β-CD, **(e)** (PEH + β-CD) physical mixture, **(f)** (PEH + β-CD) inclusion complex.

Scanning Electron Microscopy (SEM) is an exceedingly well-known technique for analyzing the surface texture and particle size of solid materials. The surface morphological structures of α-CD, (SNP + α-CD) physical mixture, (SNP + α-CD) inclusion complex, β-CD, (SNP + β-CD) physical mixture, (SNP + β-CD) inclusion complex are shown in Fig. 17 respectively. From Fig. 17 it is clear, that the morphological structures that they are totally different from each other. Similarly, the surface morphological structures of α-CD, (PEH + α-CD) physical mixture, (PEH + α-CD) inclusion complex, β-CD, (PEH + β-CD) physical mixture, (PEH + β-CD) inclusion complex are shown in Fig. 18 respectively. From Fig. 18 it is clear, that the morphological structures that they are totally different from each other. Moreover, as the complexation by α-CD and β-CD can be viewed distinctly. This is an additional evidence about the formation of inclusion complexes of SNP/PEH with α and β-CD, may support the same evident from 2D ROESY NMR analysis.

### Cytotoxic activity of the Inclusion complexes

**Figure 19:**
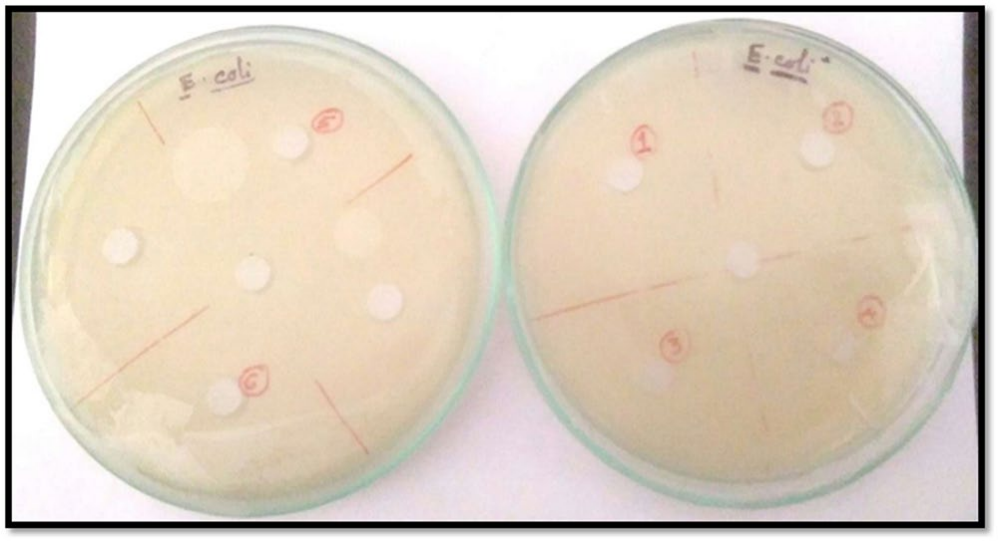
Antimicrobial activity analysis α-CD, β-CD on Gram-negative *E. coli* by Agar Cup method. No zone of inhibition is observed. Double distilled water is taken as the control. [Marker points (red) for the verified samples taken in the plates (1. SNP, 2. PEHB, 3. PEHA, 4. SNPA, 5. PEH, 6. SNPB) and Marker points (black) for the model organism taken in the plates].

**Figure 20:**
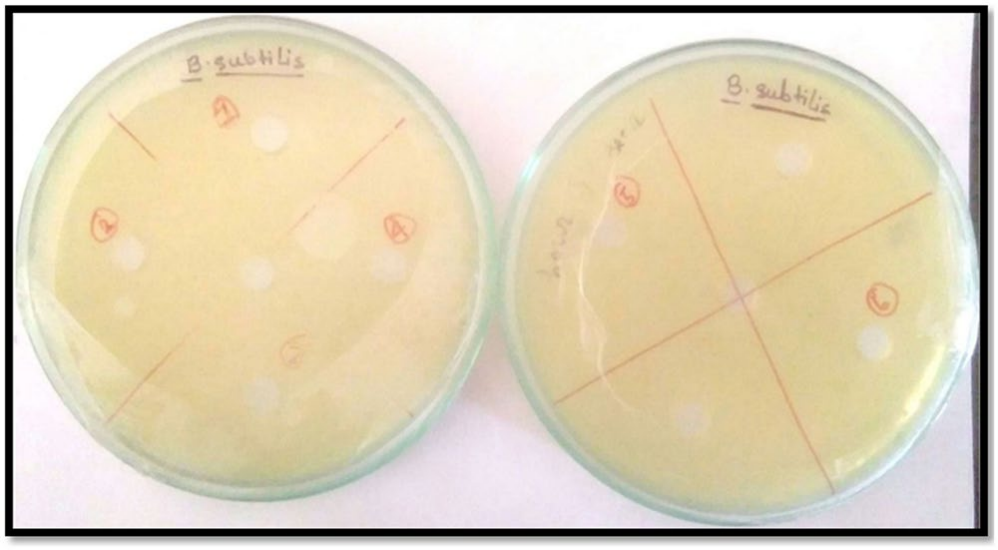
Antimicrobial activity analysis α-CD, β-CD on Gram-positive B. subtilis by Agar Cup method. No zone of inhibition is observed. Double distilled water is taken as the control. [Marker points (red) for the verified samples taken in the plates (1. SNP, 2. PEHB, 3. PEHA, 4. SNPA, 5. PEH, 6. SNPB) and Marker points (black) for the model organism taken in the plates].

No zone of inhibition is observed in case of both the gram-positive and gram-negative organisms^73,74^. There is no growth inhibition compared to control. These results suggest that ICs doesn’t have any antimicrobial activity. So, it can be said that it is non-toxic for the cells. After that cell viability assay is finished. Here, we have found that cell viability of *E. coli* is 4.6% and 9% increase in presence of [SNP + β-CD, SNP + α-CD] and [PEH + β-CD, PEH + α-CD] respectively whereas the cell viability of B. subtilis is 3.2% and 8% increase in the presence of [SNP + β-CD, SNP + α-CD] and [PEH + β-CD, PEH + α-CD] correspondingly (Figs 19, 20). These consequences indicate that cell viability is positively regulated in occurrence of these ICs (Figs 19, 20). But there is very significant increase in growth when the samples are treated with (SNP + β-CD). So, this (SNP + β-CD) is more suitable for pharmaceutically active compounds. The outcome shows that both inclusion complexes have increased the capability of SNP inhibiting cell growth rather than PEH. Particularly, SNP, complexed with beta-cyclodextrin (β-CD) show the highest cytotoxic activity resting on *E. coli* and B. subtilis; with alpha-cyclodextrin (α-CD) the cytotoxic activity is found to be rather low.

## Conclusions

The suggestion obtained from surface tension and conductometric study for the formation of 1:1 host-guest inclusion complexes of SNP and PEH with α and β-cyclodextrins are established by UV-Vis spectroscopy, spectrofluorimetry, 2D ROESY NMR spectrometry and SEM technique by analyzing surface texture of the solid inclusion complexes. The association constants obtained from all the well-established techniques dictates the stability of inclusion complexes formed and the thermodynamic parameters reveals truth about the feasibility of their formation. Removal of water molecules from the cavity of the CDs to make room for the guest molecule for accommodation while formation of inclusion complex, increases entropy of the process. Dimensional suitability being, one of the major stabilizing factor, the larger cavity size of β-CD (0.70 nm, diameter) compared to α-CD (0.56 nm, diameter), explains for the greater value of association constants and stability of the inclusion complexes formed with β-CD. The association constants, hence stability of the inclusion complexes of SNP with CDs were found more than that of the PEH. Because, -O-H group of SNP, being oriented to the para position may exert H-bonding interaction with CDs to some greater extent than that of the PEH, in which –OH group, being oriented at the meta – position can’t travel the minimum distance for the formation of H-bond with the CDs. The hydrophobic and H-bonding interactions thus stabilizes the ICs. The Cytotoxicity and Cell viability also balances for non-toxic behavior of the ICs. Thus, inclusion complexes of the recently emerging two drugs, SNP and PEH (after their banned alternatives) stabilizes SNP and PEH from their chemical modification and conveys a new approach for regulatory release to the targeted site reducing overdoses.

## Electronic supplementary material

Supporting information
