# Supplementary material for: Study to Probe Subsistence of Host-Guest Inclusion Complexes of α and β-Cyclodextrins with Biologically Potent Drugs for Safety Regulatory Dischargement
Source: Sci Rep. 2018 Aug 29;8:13031. doi: 10.1038/s41598-018-31373-x (PMC6115366; doi:10.1038/s41598-018-31373-x)
Supplement: Supplementary file 1 — Supporting information [file 41598_2018_31373_MOESM1_ESM.docx]

**SUPPORTING INFORMATION**

**Study to Probe Subsistence of Host-Guest Inclusion Complexes of α and β-Cyclodextrins with Biologically Potent Drugs for Safety Regulatory Dischargement**

^a^Biplab Rajbanshi, ^a^Subhadeep Saha, ^a^Koyeli Das, ^a^Biraj Kumar Barman, ^b^Swarnab Sengupta, ^b^Arindam Bhattacharjee, ^a^Mahendra Nath Roy*

^a^Department of Chemistry, University of North Bengal, Darjeeling – 734013, India

^b^Department of Microbiology, University of North Bengal, Darjeeling – 734013, India

*Corresponding author: Mahendra Nath Roy, Department of Chemistry, University of North Bengal, Darjeeling – 734013, E-mail: [*mahendraroy2002@yahoo.co.in*](mailto:mahendraroy2002@yahoo.co.in)

**TABLES:**

| Table S1 | Page 2 | Table S12 | Page 10 |
| --- | --- | --- | --- |
| Table S2 | Page 2 | Table S13 | Page 11 |
| Table S3 | Page 3 | Table S14 | Page 11 |
| Table S4 | Page 3 | Table S15 | Page 12 |
| Table S5 | Page 4 | Table S16 | Page 12 |
| Table S6 | Page 5 | Table S17 | Page 12 |
| Table S7 | Page 6 | Table S18 | Page 13 |
| Table S8 | Page 7 | Table S19 | Page 13 |
| Table S9 | Page 8 | Table S20 | Page 13 |
| Table S10 | Page 9 | Table S21 | Page 14 |
| Table S11 | Page 10 | Table S22-S23 | Page 15 |

**FIGURES:**

| Figure S1 | Page 18 | Figure S8 | Page 24 |
| --- | --- | --- | --- |
| Figure S2 | Page 19 | Figure S9 | Page 24 |
| Figure S3 | Page 20 | Figure S10 | Page 25 |
| Figure S4 | Page 21 | Figure S11 | Page 25 |
| Figure S5 | Page 22 | Figure S12 | Page 25 |
| Figure S6 | Page 23 | Figure S13-S18 | Page 26-28 |
| Figure S7 | Page 24 |  |  |

**TABLES:**

**Table S1**. UV-Vis spectroscopic data for the generation of Job plots of aqueous SNP+α-CD
system at 298.15 K^a^.

| **SNP + ALPHA - CYCLODEXTRIN** | | | | | | | |
| --- | --- | --- | --- | --- | --- | --- | --- |
| **SNP (mL)** | **α-CD (mL)** | **SNP (μM)** | **α-CD (μM)** | $\frac{\mathbf{[SNP]}}{\left[ \mathbf{SNP} \right]\boldsymbol{+[\alpha-CD]}}$ | **Absorbance (A)** | **ΔA** | $\frac{\boldsymbol{\Delta A\times[SNP]}}{\left[ \mathbf{SNP} \right]\boldsymbol{+[\alpha-CD]}}$ |
| 0 | 3 | 0 | 100 | 0 | 0.0000 | 0.7049 | 0.0000 |
| 0.3 | 2.7 | 10 | 90 | 0.1 | 0.0773 | 0.6276 | 0.0628 |
| 0.6 | 2.4 | 20 | 80 | 0.2 | 0.1553 | 0.5496 | 0.1099 |
| 0.9 | 2.1 | 30 | 70 | 0.3 | 0.2248 | 0.4801 | 0.1440 |
| 1.2 | 1.8 | 40 | 60 | 0.4 | 0.3057 | 0.3992 | 0.1597 |
| 1.5 | 1.5 | 50 | 50 | 0.5 | 0.3797 | 0.3252 | 0.1626 |
| 1.8 | 1.2 | 60 | 40 | 0.6 | 0.4645 | 0.2403 | 0.1442 |
| 2.1 | 0.9 | 70 | 30 | 0.7 | 0.5201 | 0.1848 | 0.1293 |
| 2.4 | 0.6 | 80 | 20 | 0.8 | 0.5830 | 0.1219 | 0.0975 |
| 2.7 | 0.3 | 90 | 10 | 0.9 | 0.6492 | 0.0557 | 0.0501 |
| 3 | 0 | 100 | 0 | 1 | 0.7049 | 0.0000 | 0.0000 |

^a^Standard uncertainties in temperature *u* are: *u*(T) = ±0.01 K.

**Table S2**. UV-Vis spectroscopic data for the generation of Job plots of aqueous SNP+β-CD
system at 298.15 K^a^.

| **SNP + BETA - CYCLODEXTRIN** | | | | | | | |
| --- | --- | --- | --- | --- | --- | --- | --- |
| **SNP (mL)** | **β-CD (mL)** | **SNP (μM)** | **β-CD (μM)** | $\frac{\mathbf{[SNP]}}{\left[ \mathbf{SNP} \right]\boldsymbol{+[\beta-CD]}}$ | **Absorbance (A)** | **ΔA** | $\frac{\boldsymbol{\Delta A\times[SNP]}}{\left[ \mathbf{SNP} \right]\boldsymbol{+[\beta-CD]}}$ |
| 0 | 3 | 0 | 100 | 0 | 0.0000 | 0.7049 | 0.0000 |
| 0.3 | 2.7 | 10 | 90 | 0.1 | 0.0434 | 0.6614 | 0.0661 |
| 0.6 | 2.4 | 20 | 80 | 0.2 | 0.1245 | 0.5804 | 0.1161 |
| 0.9 | 2.1 | 30 | 70 | 0.3 | 0.2146 | 0.4903 | 0.1471 |
| 1.2 | 1.8 | 40 | 60 | 0.4 | 0.2716 | 0.4332 | 0.1733 |
| 1.5 | 1.5 | 50 | 50 | 0.5 | 0.3499 | 0.3550 | 0.1775 |
| 1.8 | 1.2 | 60 | 40 | 0.6 | 0.4266 | 0.2783 | 0.1670 |
| 2.1 | 0.9 | 70 | 30 | 0.7 | 0.4967 | 0.2082 | 0.1457 |
| 2.4 | 0.6 | 80 | 20 | 0.8 | 0.5709 | 0.1340 | 0.1072 |
| 2.7 | 0.3 | 90 | 10 | 0.9 | 0.6290 | 0.0759 | 0.0683 |
| 3 | 0 | 100 | 0 | 1 | 0.7049 | 0.0000 | 0.0000 |

**Table S3**. UV-Vis spectroscopic data for the generation of Job plots of aqueous PEH+α-CD
system at 298.15 K^a^.

| **PEH + ALPHA - CYCLODEXTRIN** | | | | | | | |
| --- | --- | --- | --- | --- | --- | --- | --- |
| **PEH (mL)** | **α-CD (mL)** | **PEH (μM)** | **α-CD (μM)** | $\frac{\mathbf{[PEH]}}{\left[ \mathbf{PEH} \right]\boldsymbol{+[\alpha-CD]}}$ | **Absorbance (A)** | **ΔA** | $\frac{\boldsymbol{\Delta A\times[PEH]}}{\left[ \mathbf{PEH} \right]\boldsymbol{+[\alpha-CD]}}$ |
| 0 | 3 | 0 | 100 | 0 | 0.0000 | 0.6606 | 0.0000 |
| 0.3 | 2.7 | 10 | 90 | 0.1 | 0.0680 | 0.5927 | 0.0593 |
| 0.6 | 2.4 | 20 | 80 | 0.2 | 0.1376 | 0.5230 | 0.1046 |
| 0.9 | 2.1 | 30 | 70 | 0.3 | 0.2052 | 0.4554 | 0.1366 |
| 1.2 | 1.8 | 40 | 60 | 0.4 | 0.2831 | 0.3775 | 0.1510 |
| 1.5 | 1.5 | 50 | 50 | 0.5 | 0.3432 | 0.3175 | 0.1587 |
| 1.8 | 1.2 | 60 | 40 | 0.6 | 0.4107 | 0.2499 | 0.1500 |
| 2.1 | 0.9 | 70 | 30 | 0.7 | 0.4743 | 0.1864 | 0.1305 |
| 2.4 | 0.6 | 80 | 20 | 0.8 | 0.5404 | 0.1202 | 0.0962 |
| 2.7 | 0.3 | 90 | 10 | 0.9 | 0.6092 | 0.0515 | 0.0463 |
| 3 | 0 | 100 | 0 | 1 | 0.6606 | 0.0000 | 0.0000 |

**Table S4**. UV-Vis spectroscopic data for the generation of Job plots of aqueous SNP+β-CD
system at 298.15 K^a^.

| **PEH + BETA - CYCLODEXTRIN** | | | | | | | |
| --- | --- | --- | --- | --- | --- | --- | --- |
| **PEH (mL)** | **β-CD (mL)** | **PEH (μM)** | **β-CD (μM)** | $\frac{\mathbf{[PEH]}}{\left[ \mathbf{PEH} \right]\boldsymbol{+[\beta-CD]}}$ | **Absorbance (A)** | **ΔA** | $\frac{\boldsymbol{\Delta A\times[PEH]}}{\left[ \mathbf{PEH} \right]\boldsymbol{+[\beta-CD]}}$ |
| 0 | 3 | 0 | 100 | 0 | 0.0000 | 0.6606 | 0.0000 |
| 0.3 | 2.7 | 10 | 90 | 0.1 | 0.0398 | 0.6208 | 0.0621 |
| 0.6 | 2.4 | 20 | 80 | 0.2 | 0.1119 | 0.5487 | 0.1097 |
| 0.9 | 2.1 | 30 | 70 | 0.3 | 0.1827 | 0.4780 | 0.1434 |
| 1.2 | 1.8 | 40 | 60 | 0.4 | 0.2511 | 0.4096 | 0.1638 |
| 1.5 | 1.5 | 50 | 50 | 0.5 | 0.3264 | 0.3342 | 0.1671 |
| 1.8 | 1.2 | 60 | 40 | 0.6 | 0.3865 | 0.2741 | 0.1645 |
| 2.1 | 0.9 | 70 | 30 | 0.7 | 0.4695 | 0.1911 | 0.1338 |
| 2.4 | 0.6 | 80 | 20 | 0.8 | 0.5291 | 0.1315 | 0.1052 |
| 2.7 | 0.3 | 90 | 10 | 0.9 | 0.5904 | 0.0702 | 0.0632 |
| 3 | 0 | 100 | 0 | 1 | 0.6606 | 0.0000 | 0.0000 |

**Table S5**. Data for surface tension of aqueous (SNP+α-CD) and (SNP+β-CD) systems at 298.15 K^a^.

| **SNP+α-CD** | | | |
| --- | --- | --- | --- |
| **SNP (mL)** | **α-CD (mL)** | **Concentration of α-CD (mM)** | **Surface tension (mNm^-1^)** |
| 10 | 0 | 0.0000 | 62.2 |
| 10 | 1 | 0.9091 | 63.2 |
| 10 | 2 | 1.6667 | 64.4 |
| 10 | 3 | 2.3077 | 65.3 |
| 10 | 4 | 2.8571 | 66.2 |
| 10 | 5 | 3.3333 | 66.9 |
| 10 | 6 | 3.7500 | 67.8 |
| 10 | 7 | 4.1176 | 68.5 |
| 10 | 8 | 4.4444 | 69.0 |
| 10 | 9 | 4.7368 | 69.5 |
| 10 | 10 | 5.0000 | 70.0 |
| 10 | 11 | 5.2381 | 70.2 |
| 10 | 12 | 5.4545 | 70.3 |
| 10 | 13 | 5.6522 | 70.4 |
| 10 | 14 | 5.8333 | 70.5 |
| 10 | 15 | 6.0000 | 70.6 |
| 10 | 16 | 6.1538 | 70.7 |
| 10 | 17 | 6.2963 | 70.8 |
| 10 | 18 | 6.4286 | 70.9 |
| 10 | 19 | 6.5517 | 71.0 |
| 10 | 20 | 6.6667 | 71.0 |
| **SNP+β-CD** | | | |
| **SNP (mL)** | **β-CD (mL)** | **Concentration of β-CD (mM)** | **Surface tension (mNm^-1^)** |
| 10 | 0 | 0.0000 | 62.2 |
| 10 | 1 | 0.9091 | 63.5 |
| 10 | 2 | 1.6667 | 64.7 |
| 10 | 3 | 2.3077 | 65.7 |
| 10 | 4 | 2.8571 | 66.5 |
| 10 | 5 | 3.3333 | 67.3 |
| 10 | 6 | 3.7500 | 68.1 |
| 10 | 7 | 4.1176 | 68.8 |
| 10 | 8 | 4.4444 | 69.3 |
| 10 | 9 | 4.7368 | 69.8 |
| 10 | 10 | 5.0000 | 70.3 |
| 10 | 11 | 5.2381 | 70.5 |
| 10 | 12 | 5.4545 | 70.6 |
| 10 | 13 | 5.6522 | 70.7 |
| 10 | 14 | 5.8333 | 70.8 |
| 10 | 15 | 6.0000 | 70.9 |
| 10 | 16 | 6.1538 | 71.0 |
| 10 | 17 | 6.2963 | 71.1 |
| 10 | 18 | 6.4286 | 71.2 |
| 10 | 19 | 6.5517 | 71.3 |
| 10 | 20 | 6.6667 | 71.3 |

**Table S6**. Data for surface tension of aqueous (PEH+α-CD) and (PEH+β-CD) systems at 298.15 K^a^.

| **PEH+α-CD** | | | |
| --- | --- | --- | --- |
| **PEH (mL)** | **α-CD (mL)** | **Concentration of α-CD (mM)** | **Surface tension (mNm^-1^)** |
| 10 | 0 | 0.0000 | 52.6 |
| 10 | 1 | 0.9091 | 55.6 |
| 10 | 2 | 1.6667 | 58.4 |
| 10 | 3 | 2.3077 | 60.9 |
| 10 | 4 | 2.8571 | 62.7 |
| 10 | 5 | 3.3333 | 64.6 |
| 10 | 6 | 3.7500 | 65.9 |
| 10 | 7 | 4.1176 | 67.3 |
| 10 | 8 | 4.4444 | 68.4 |
| 10 | 9 | 4.7368 | 69.5 |
| 10 | 10 | 5.0000 | 70.3 |
| 10 | 11 | 5.2381 | 70.4 |
| 10 | 12 | 5.4545 | 70.5 |
| 10 | 13 | 5.6522 | 70.6 |
| 10 | 14 | 5.8333 | 70.7 |
| 10 | 15 | 6.0000 | 70.8 |
| 10 | 16 | 6.1538 | 70.9 |
| 10 | 17 | 6.2963 | 71.0 |
| 10 | 18 | 6.4286 | 71.1 |
| 10 | 19 | 6.5517 | 71.2 |
| 10 | 20 | 6.6667 | 71.2 |
| **PEH+β-CD** | | | |
| **PEH (mL)** | **β-CD (mL)** | **Concentration of β-CD (mM)** | **Surface tension (mNm^-1^)** |
| 10 | 0 | 0.0000 | 52.6 |
| 10 | 1 | 0.9091 | 55.9 |
| 10 | 2 | 1.6667 | 58.7 |
| 10 | 3 | 2.3077 | 61.2 |
| 10 | 4 | 2.8571 | 63.1 |
| 10 | 5 | 3.3333 | 64.9 |
| 10 | 6 | 3.7500 | 66.2 |
| 10 | 7 | 4.1176 | 67.6 |
| 10 | 8 | 4.4444 | 68.7 |
| 10 | 9 | 4.7368 | 69.8 |
| 10 | 10 | 5.0000 | 70.6 |
| 10 | 11 | 5.2381 | 70.7 |
| 10 | 12 | 5.4545 | 70.8 |
| 10 | 13 | 5.6522 | 70.9 |
| 10 | 14 | 5.8333 | 71.0 |
| 10 | 15 | 6.0000 | 71.1 |
| 10 | 16 | 6.1538 | 71.2 |
| 10 | 17 | 6.2963 | 71.3 |
| 10 | 18 | 6.4286 | 71.4 |
| 10 | 19 | 6.5517 | 71.4 |
| 10 | 20 | 6.6667 | 71.5 |

**Table S7**. UV-vis spectroscopic data for the Benesi-Hildebrand double reciprocal plot of (SNP+α-CD) system at 298.15 to 308.15 K^a^.

| **Temp (K^a^)** | **SNP**  **(μM)** | **α-CD**  **(μM)** | **Ao** | **A** | **ΔA** | **1/[α-CD]**  **(M^-1^)** | **1/ΔA** | **Intercept** | **Slope** | **Ka**  **(M-^1^×10^-3^)** |
| --- | --- | --- | --- | --- | --- | --- | --- | --- | --- | --- |
| 298.15 | 50 | 20 | 0.3524 | 0.3659 | 0.0135 | 0.0500 | 74.3494 | 4.0631 | 1429.4 | 2.84 |
|  | 50 | 30 |  | 0.3715 | 0.0191 | 0.0333 | 52.4384 |  |  |  |
|  | 50 | 40 |  | 0.3763 | 0.0238 | 0.0250 | 41.9639 |  |  |  |
|  | 50 | 50 |  | 0.3829 | 0.0304 | 0.0200 | 32.8515 |  |  |  |
|  | 50 | 60 |  | 0.3875 | 0.0351 | 0.0167 | 28.5063 |  |  |  |
|  | 50 | 70 |  | 0.3953 | 0.0429 | 0.0143 | 23.3209 |  |  |  |
|  | 50 | 80 |  | 0.4011 | 0.0487 | 0.0125 | 20.5550 |  |  |  |
| 303.15 | 50 | 20 | 0.3524 | 0.3651 | 0.0127 | 0.0500 | 78.9889 | 3.4735 | 1536.2 | 2.26 |
|  | 50 | 30 |  | 0.3704 | 0.0180 | 0.0333 | 55.6793 |  |  |  |
|  | 50 | 40 |  | 0.3751 | 0.0227 | 0.0250 | 44.1112 |  |  |  |
|  | 50 | 50 |  | 0.3815 | 0.0291 | 0.0200 | 34.4234 |  |  |  |
|  | 50 | 60 |  | 0.3867 | 0.0343 | 0.0167 | 29.1545 |  |  |  |
|  | 50 | 70 |  | 0.3938 | 0.0414 | 0.0143 | 24.1501 |  |  |  |
|  | 50 | 80 |  | 0.3985 | 0.0461 | 0.0125 | 21.7004 |  |  |  |
| 308.15 | 50 | 20 | 0.3524 | 0.3645 | 0.0121 | 0.0500 | 82.9187 | 2.8374 | 1630.9 | 1.74 |
|  | 50 | 30 |  | 0.3694 | 0.0170 | 0.0333 | 58.9623 |  |  |  |
|  | 50 | 40 |  | 0.3745 | 0.0221 | 0.0250 | 45.3104 |  |  |  |
|  | 50 | 50 |  | 0.3807 | 0.0283 | 0.0200 | 35.3982 |  |  |  |
|  | 50 | 60 |  | 0.3858 | 0.0334 | 0.0167 | 29.9401 |  |  |  |
|  | 50 | 70 |  | 0.3927 | 0.0403 | 0.0143 | 24.8092 |  |  |  |
|  | 50 | 80 |  | 0.3965 | 0.0441 | 0.0125 | 22.6850 |  |  |  |

^a^Standard uncertainties in temperature *u* are: *u*(T) = ±0.01 K.

**Table S8**. UV-vis spectroscopic data for the Benesi-Hildebrand double reciprocal plot of (SNP+β-CD) systems at 298.15 to 308.15 K^a^.

| **Temp (K^a^)** | **SNP (μM)** | **β-CD**  **(μM)** | **Ao** | **A** | **ΔA** | **1/[β-CD]**  **(M^-1^)** | **1/ΔA** | **Intercept** | **Slope** | **Ka**  **(M^-1^×10^-3^)** |
| --- | --- | --- | --- | --- | --- | --- | --- | --- | --- | --- |
| 298.15 | 50 | 20 | 0.3524 | 0.3666 | 0.0142 | 0.0500 | 70.6714 | 4.6812 | 1341.6 | 3.82 |
|  | 50 | 30 |  | 0.3723 | 0.0199 | 0.0333 | 50.3018 |  |  |  |
|  | 50 | 40 |  | 0.3775 | 0.0251 | 0.0250 | 39.8724 |  |  |  |
|  | 50 | 50 |  | 0.3840 | 0.0315 | 0.0200 | 31.7158 |  |  |  |
|  | 50 | 60 |  | 0.3889 | 0.0365 | 0.0167 | 27.4198 |  |  |  |
|  | 50 | 70 |  | 0.3961 | 0.0437 | 0.0143 | 22.8833 |  |  |  |
|  | 50 | 80 |  | 0.4015 | 0.0491 | 0.0125 | 20.3666 |  |  |  |
| 303.15 | 50 | 20 | 0.3524 | 0.3656 | 0.0131 | 0.0500 | 76.0514 | 4.188 | 1453.5 | 2.88 |
|  | 50 | 30 |  | 0.3712 | 0.0188 | 0.0333 | 53.3049 |  |  |  |
|  | 50 | 40 |  | 0.3762 | 0.0238 | 0.0250 | 42.0698 |  |  |  |
|  | 50 | 50 |  | 0.3826 | 0.0302 | 0.0200 | 33.1675 |  |  |  |
|  | 50 | 60 |  | 0.3878 | 0.0354 | 0.0167 | 28.2486 |  |  |  |
|  | 50 | 70 |  | 0.3938 | 0.0414 | 0.0143 | 24.1721 |  |  |  |
|  | 50 | 80 |  | 0.3979 | 0.0455 | 0.0125 | 21.9925 |  |  |  |
| 308.15 | 50 | 20 | 0.3524 | 0.3651 | 0.0127 | 0.0500 | 78.9889 | 3.6968 | 1520.5 | 2.28 |
|  | 50 | 30 |  | 0.3707 | 0.0183 | 0.0333 | 54.7645 |  |  |  |
|  | 50 | 40 |  | 0.3755 | 0.0231 | 0.0250 | 43.3463 |  |  |  |
|  | 50 | 50 |  | 0.3817 | 0.0293 | 0.0200 | 34.1880 |  |  |  |
|  | 50 | 60 |  | 0.3869 | 0.0345 | 0.0167 | 28.9855 |  |  |  |
|  | 50 | 70 |  | 0.3931 | 0.0407 | 0.0143 | 24.5654 |  |  |  |
|  | 50 | 80 |  | 0.3974 | 0.0450 | 0.0125 | 22.2311 |  |  |  |

^a^Standard uncertainties in temperature *u* are: *u*(T) = ±0.01 K.

**Table S9**. UV-vis spectroscopic data for the Benesi-Hildebrand double reciprocal plot of (PEH+α-CD) system at 298.15 to 308.15 K^a^.

| **Temp (K^a^)** | **PEH (μM)** | **α-CD**  **(μM)** | **Ao** | **A** | **ΔA** | **1/[α-CD]**  **(M^-1^)** | **1/ΔA** | **Intercept** | **Slope** | **Ka**  **(M^-1^×10^-3^)** |
| --- | --- | --- | --- | --- | --- | --- | --- | --- | --- | --- |
| 298.15 | 50 | 20 | 0.3303 | 0.3437 | 0.0134 | 0.0500 | 74.9064 | 3.1053 | 1453.1 | 2.14 |
|  | 50 | 30 |  | 0.3491 | 0.0188 | 0.0333 | 53.135 |  |  |  |
|  | 50 | 40 |  | 0.3555 | 0.0252 | 0.0250 | 39.7456 |  |  |  |
|  | 50 | 50 |  | 0.3618 | 0.0315 | 0.0200 | 31.746 |  |  |  |
|  | 50 | 60 |  | 0.3676 | 0.0373 | 0.0167 | 26.8312 |  |  |  |
|  | 50 | 70 |  | 0.3720 | 0.0417 | 0.0143 | 23.9866 |  |  |  |
|  | 50 | 80 |  | 0.3779 | 0.0476 | 0.0125 | 21.0128 |  |  |  |
| 303.15 | 50 | 20 | 0.3303 | 0.3430 | 0.0127 | 0.0500 | 79.0514 | 2.7682 | 1546.3 | 1.79 |
|  | 50 | 30 |  | 0.3481 | 0.0178 | 0.0333 | 56.1167 |  |  |  |
|  | 50 | 40 |  | 0.3542 | 0.0239 | 0.0250 | 41.9111 |  |  |  |
|  | 50 | 50 |  | 0.3603 | 0.0300 | 0.0200 | 33.3333 |  |  |  |
|  | 50 | 60 |  | 0.3661 | 0.0358 | 0.0167 | 27.9564 |  |  |  |
|  | 50 | 70 |  | 0.3704 | 0.0401 | 0.0143 | 24.9439 |  |  |  |
|  | 50 | 80 |  | 0.3764 | 0.0461 | 0.0125 | 21.6967 |  |  |  |
| 308.15 | 50 | 20 | 0.3303 | 0.3423 | 0.0120 | 0.0500 | 83.682 | 2.4393 | 1647.2 | 1.48 |
|  | 50 | 30 |  | 0.3471 | 0.0168 | 0.0333 | 59.453 |  |  |  |
|  | 50 | 40 |  | 0.3531 | 0.0228 | 0.0250 | 43.9367 |  |  |  |
|  | 50 | 50 |  | 0.3589 | 0.0286 | 0.0200 | 34.965 |  |  |  |
|  | 50 | 60 |  | 0.3646 | 0.0343 | 0.0167 | 29.18 |  |  |  |
|  | 50 | 70 |  | 0.3688 | 0.0385 | 0.0143 | 25.9808 |  |  |  |
|  | 50 | 80 |  | 0.3741 | 0.0438 | 0.0125 | 22.8363 |  |  |  |

^a^Standard uncertainties in temperature *u* are: *u*(T) = ±0.01 K.

**Table S10**. UV-vis spectroscopic data for the Benesi-Hildebrand double reciprocal plot of (PEH+β-CD) systems at 298.15 to 308.15 K^a^.

| **Temp (K^a^)** | **PEH (μM)** | **β-CD**  **(μM)** | **Ao** | **A** | **ΔA** | **1/[β-CD]**  **(M^-1^)** | **1/ΔA** | **Intercept** | **Slope** | **Ka**  **(M^-1^×10^-3^)** |
| --- | --- | --- | --- | --- | --- | --- | --- | --- | --- | --- |
| 298.15 | 50 | 20 | 0.3303 | 0.3444 | 0.0141 | 0.0500 | 71.1744 | 4.037 | 1360.8 | 2.97 |
|  | 50 | 30 |  | 0.3499 | 0.0196 | 0.0333 | 50.9684 |  |  |  |
|  | 50 | 40 |  | 0.3563 | 0.0260 | 0.0250 | 38.5208 |  |  |  |
|  | 50 | 50 |  | 0.3626 | 0.0323 | 0.0200 | 30.9598 |  |  |  |
|  | 50 | 60 |  | 0.3686 | 0.0383 | 0.0167 | 26.1301 |  |  |  |
|  | 50 | 70 |  | 0.3729 | 0.0426 | 0.0143 | 23.4797 |  |  |  |
|  | 50 | 80 |  | 0.3784 | 0.0481 | 0.0125 | 20.7943 |  |  |  |
| 303.15 | 50 | 20 | 0.3303 | 0.3434 | 0.0131 | 0.0500 | 76.6284 | 3.3425 | 1481.7 | 2.26 |
|  | 50 | 30 |  | 0.3487 | 0.0184 | 0.0333 | 54.2888 |  |  |  |
|  | 50 | 40 |  | 0.3549 | 0.0246 | 0.0250 | 40.7166 |  |  |  |
|  | 50 | 50 |  | 0.3611 | 0.0308 | 0.0200 | 32.4675 |  |  |  |
|  | 50 | 60 |  | 0.3667 | 0.0364 | 0.0167 | 27.4801 |  |  |  |
|  | 50 | 70 |  | 0.3716 | 0.0413 | 0.0143 | 24.2189 |  |  |  |
|  | 50 | 80 |  | 0.3755 | 0.0452 | 0.0125 | 22.1288 |  |  |  |
| 308.15 | 50 | 20 | 0.3303 | 0.3428 | 0.0125 | 0.0500 | 80.3213 | 2.8083 | 1573.5 | 1.79 |
|  | 50 | 30 |  | 0.3477 | 0.0174 | 0.0333 | 57.4053 |  |  |  |
|  | 50 | 40 |  | 0.3539 | 0.0236 | 0.0250 | 42.4448 |  |  |  |
|  | 50 | 50 |  | 0.3597 | 0.0294 | 0.0200 | 34.0136 |  |  |  |
|  | 50 | 60 |  | 0.3655 | 0.0352 | 0.0167 | 28.4333 |  |  |  |
|  | 50 | 70 |  | 0.3702 | 0.0399 | 0.0143 | 25.0689 |  |  |  |
|  | 50 | 80 |  | 0.3752 | 0.0449 | 0.0125 | 22.2767 |  |  |  |

^a^Standard uncertainties in temperature *u* are: *u*(T) = ±0.01 K.

**Table S11**. Spectro-fluorimetric data for the Benesi-Hildebrand double reciprocal plot of (SNP+α-CD) system at 298.15 K^a^.

| **SNP**  **(μM)** | **α-CD**  **(μM)** | **$\text{I}_{0}$** | **$\text{I}$** | **$\text{I-I}_{0}$** | **1/[α-CD]**  **(M^-1^)** | **1/ΔI**  **(×10^5^)** | **Intercept**  **(×10^6^)** | **Slope**  **(×10^10^)** | **Ka**  **(M^-1^×10^-3^)** |
| --- | --- | --- | --- | --- | --- | --- | --- | --- | --- |
| 50 | 20 | 989842.6 | 1021722.0 | 31879.4 | 0.0500 | 3.1368 | 1.70 | 5.93 | 2.87 |
| 50 | 30 |  | 1036798.0 | 46955.4 | 0.0333 | 2.1297 |  |  |  |
| 50 | 40 |  | 1049428.0 | 59585.4 | 0.0250 | 1.6783 |  |  |  |
| 50 | 50 |  | 1064623.0 | 74780.4 | 0.0200 | 1.3372 |  |  |  |
| 50 | 60 |  | 1073962.6 | 84120.0 | 0.0167 | 1.1888 |  |  |  |
| 50 | 70 |  | 1088841.6 | 98999.0 | 0.0143 | 1.0101 |  |  |  |
| 50 | 80 |  | 1101249.0 | 111406.4 | 0.0125 | 0.8976 |  |  |  |

^a^Standard uncertainties in temperature *u* are: *u*(T) = ±0.01 K.

**Table S12**. Spectro-fluorimetric data for the Benesi-Hildebrand double reciprocal plot of (SNP+β-CD) system at 298.15 K^a^.

| **SNP**  **(μM)** | **β-CD**  **(μM)** | **$\text{I}_{0}$** | **$\text{I}$** | **$\text{I-I}_{0}$** | **1/[β-CD]**  **(M^-1^)** | **1/ΔI**  **(×10^5^)** | **Intercept**  **(×10^6^)** | **Slope**  **(×10^10^)** | **Ka**  **(M^-1^×10^-3^)** |
| --- | --- | --- | --- | --- | --- | --- | --- | --- | --- |
| 50 | 20 | 989842.6 | 1032096 | 42253.4 | 0.0500 | 2.3667 | 1.64 | 4.40 | 3.73 |
| 50 | 30 |  | 1050436 | 60593.4 | 0.0333 | 1.6503 |  |  |  |
| 50 | 40 |  | 1071194 | 81351.4 | 0.0250 | 1.2292 |  |  |  |
| 50 | 50 |  | 1086066 | 96223.4 | 0.0200 | 1.0392 |  |  |  |
| 50 | 60 |  | 1102000 | 112157.4 | 0.0167 | 0.8916 |  |  |  |
| 50 | 70 |  | 1115745 | 125902.4 | 0.0143 | 0.7943 |  |  |  |
| 50 | 80 |  | 1125567 | 135724.4 | 0.0125 | 0.7368 |  |  |  |

^a^Standard uncertainties in temperature *u* are: *u*(T) = ±0.01 K.

**Table S13**. Spectro-fluorimetric data for the Benesi-Hildebrand double reciprocal plot of (PEH+α-CD) system at 298.15 K^a^.

| **PEH (μM)** | **α-CD**  **(μM)** | **$\text{I}_{0}$** | **$\text{I}$** | **$\text{I-I}_{0}$** | **1/[α-CD]**  **(M^-1^)** | **1/ΔI**  **(×10^5^)** | **Intercept**  **(×10^6^)** | **Slope**  **(×10^10^)** | **Ka**  **(M^-1^×10^-3^)** |
| --- | --- | --- | --- | --- | --- | --- | --- | --- | --- |
| 50 | 20 | 1303230 | 1333970 | 30740 | 0.0500 | 3.2531 | 1.38 | 6.23 | 2.21 |
| 50 | 30 |  | 1349246 | 46016 | 0.0333 | 2.1732 |  |  |  |
| 50 | 40 |  | 1360816 | 57586 | 0.0250 | 1.7365 |  |  |  |
| 50 | 50 |  | 1374011 | 70781 | 0.0200 | 1.4128 |  |  |  |
| 50 | 60 |  | 1387350 | 84120 | 0.0167 | 1.1888 |  |  |  |
| 50 | 70 |  | 1402229 | 98999 | 0.0143 | 1.0101 |  |  |  |
| 50 | 80 |  | 1416037 | 112807 | 0.0125 | 0.8865 |  |  |  |

^a^Standard uncertainties in temperature *u* are: *u*(T) = ±0.01 K.

**Table S14**. Spectro-fluorimetric data for the Benesi-Hildebrand double reciprocal plot of (PEH+β-CD) system at 298.15 K^a^.

| **PEH (μM)** | **β-CD**  **(μM)** | **$\text{I}_{0}$** | **$\text{I}$** | **$\text{I-I}_{0}$** | **1/[β-CD]**  **(M^-1^)** | **1/ΔI**  **(×10^5^)** | **Intercept**  **(×10^6^)** | **Slope**  **(×10^10^)** | **Ka**  **(M^-1^×10^-3^)** |
| --- | --- | --- | --- | --- | --- | --- | --- | --- | --- |
| 50 | 20 | 1303230 | 1340002 | 36772 | 0.0500 | 2.7195 | 1.48 | 5.09 | 2.91 |
| 50 | 30 |  | 1358291 | 55061 | 0.0333 | 1.8162 |  |  |  |
| 50 | 40 |  | 1375178 | 71948 | 0.0250 | 1.3899 |  |  |  |
| 50 | 50 |  | 1391725 | 88495 | 0.0200 | 1.1300 |  |  |  |
| 50 | 60 |  | 1399947 | 96717 | 0.0167 | 1.0339 |  |  |  |
| 50 | 70 |  | 1416229 | 112999 | 0.0143 | 0.8850 |  |  |  |
| 50 | 80 |  | 1428037 | 124807 | 0.0125 | 0.8012 |  |  |  |

^a^Standard uncertainties in temperature *u* are: *u*(T) = ±0.01 K.

**Table S15**. Data of the van’t Hoff equation for calculation of thermodynamic
parameters ΔH^o^, ΔS^o^ and ΔG^0^ of different (SNP+α-CD) and (SNP+β-CD) inclusion complexes.

| **HOST** | **T(K^a^)** | **1/T** | **Ka**  **(M^-1^×10^-3^)** | **lnKa** | **Slope** | **Intercept** | **ΔH^0^**  **(KJ mol^-1^)** | **ΔS^0^**  **(J mol^-1^ K^-1^)** | **ΔG^0^**  **(KJ mol^-1^)** |
| --- | --- | --- | --- | --- | --- | --- | --- | --- | --- |
| α-CD | 303.15 | 0.0033 | 2.84 | 7.9527 | 4657.0 | -7.4026 | -38.72 | -61.55 | -20.37 |
|  | 308.15 | 0.0032 | 2.26 | 7.7236 |  |  |  |  |  |
|  | 313.15 | 0.0032 | 1.74 | 7.4619 |  |  |  |  |  |
| β-CD | 303.15 | 0.0033 | 3.82 | 8.2469 | 4908.7 | -7.9513 | -40.81 | -66.11 | -21.1 |
|  | 308.15 | 0.0032 | 2.88 | 7.9663 |  |  |  |  |  |
|  | 313.15 | 0.0032 | 2.28 | 7.7300 |  |  |  |  |  |

**Table S16**. Data of the van’t Hoff equation for calculation of thermodynamic
parameters ΔH^o^, ΔS^o^ and ΔG^0^ of different (PEH+α-CD) and (PEH+β-CD) inclusion complexes.

| **HOST** | **T(K^a^)** | **1/T** | **Ka**  **(M^-1^×10^-3^)** | **lnKa** | **Slope** | **Intercept** | **ΔH^0^**  **(KJ mol^-1^)** | **ΔS^0^**  **(J mol^-1^ K^-1^)** | **ΔG^0^**  **(KJ mol^-1^)** |
| --- | --- | --- | --- | --- | --- | --- | --- | --- | --- |
| α-CD | 303.15 | 0.0033 | 2.14 | 7.6671 | 3480.6 | -3.8114 | -28.94 | -31.69 | -19.49 |
|  | 308.15 | 0.0032 | 1.79 | 7.4902 |  |  |  |  |  |
|  | 313.15 | 0.0032 | 1.48 | 3.3004 |  |  |  |  |  |
| β-CD | 303.15 | 0.0033 | 2.97 | 7.9958 | 4829.2 | -7.9393 | -40.15 | -66.01 | -20.47 |
|  | 308.15 | 0.0032 | 2.26 | 7.7216 |  |  |  |  |  |
|  | 313.15 | 0.0032 | 1.79 | 7.4872 |  |  |  |  |  |

**Table S17**. Data of the van’t Hoff equation for calculation of thermodynamic
parameters ΔH^θ0^, ΔS^θ0^ and ΔG^θ0^ of different (SNP+α-CD) and (SNP+β-CD) inclusion complexes.

| **HOST** | **T(K^a^)** | **1/T** | **$\text{K}_{\text{a}}^{\text{θ}}$**  **(M^-1^×10^-3^)** | **ln$\text{K}_{\text{a}}^{\text{θ}}$** | **Slope** | **Intercept** | **ΔH^θ0^**  **(KJ mol^-1^)** | **ΔS^θ0^**  **(J mol^-1^ K^-1^)** | **ΔG^θ0^**  **(KJ mol^-1^)** |
| --- | --- | --- | --- | --- | --- | --- | --- | --- | --- |
| α-CD | 303.15 | 0.0033 | 2.82 | 7.9445 | 5203.0 | -9.2162 | -43.26 | -76.63 | -20.41 |
|  | 308.15 | 0.0032 | 2.15 | 7.6732 |  |  |  |  |  |
|  | 313.15 | 0.0032 | 1.63 | 7.3963 |  |  |  |  |  |
| β-CD | 303.15 | 0.0033 | 3.41 | 8.1345 | 5817.1 | -11.06 | -48.37 | -91.96 | -20.95 |
|  | 308.15 | 0.0032 | 2.46 | 7.8079 |  |  |  |  |  |
|  | 313.15 | 0.0032 | 1.85 | 7.5219 |  |  |  |  |  |

**Table S18**. Data of the van’t Hoff equation for calculation of thermodynamic
parameters ΔH^θ0^, ΔS^θ0^ and ΔG^θ0^ of different (PEH+α-CD) and (PEH+β-CD) inclusion complexes.

| **HOST** | **T(K^a^)** | **1/T** | **$\text{K}_{\text{a}}^{\text{θ}}$**  **(M^-1^×10^-3^)** | **ln$\text{K}_{\text{a}}^{\text{θ}}$** | **Slope** | **Intercept** | **ΔH^θ0^**  **(KJ mol^-1^)** | **ΔS^θ0^**  **(J mol^-1^ K^-1^)** | **ΔG^θ0^**  **(KJ mol^-1^)** |
| --- | --- | --- | --- | --- | --- | --- | --- | --- | --- |
| α-CD | 303.15 | 0.0033 | 2.07 | 7.6353 | 3,710.0 | -4.5946 | -30.85 | -38.20 | -19.46 |
|  | 308.15 | 0.0032 | 1.74 | 7.4616 |  |  |  |  |  |
|  | 313.15 | 0.0032 | 1.40 | 7.2442 |  |  |  |  |  |
| β-CD | 303.15 | 0.0033 | 2.71 | 7.9047 | 4,428.7 | -6.7102 | -36.82 | -55.79 | -20.19 |
|  | 308.15 | 0.0032 | 2.10 | 7.6497 |  |  |  |  |  |
|  | 313.15 | 0.0032 | 1.70 | 7.4384 |  |  |  |  |  |

**Table S19**. Data of the van’t Hoff equation for calculation of thermodynamic
parameters ΔH^C0^, ΔS^C0^ and ΔG^C0^of different (SNP+α-CD) and (SNP+β-CD) inclusion complexes.

| **HOST** | **T(K^a^)** | **1/T** | **$\text{K}_{\text{a}}^{C0}$**  **(M^-1^×10^-3^)** | **ln$\text{K}_{\text{a}}^{C0}$** | **Slope** | **Intercept** | **ΔH^C0^**  **(KJ mol^-1^)** | **ΔS^C0^**  **(J mol^-1^ K^-1^)** | **ΔG^C0^**  **(KJ mol^-1^)** |
| --- | --- | --- | --- | --- | --- | --- | --- | --- | --- |
| α-CD | 303.15 | 0.0033 | 2.80 | 7.9374 | 4961.5 | -8.4239 | -41.25 | -70.04 | -20.37 |
|  | 308.15 | 0.0032 | 2.18 | 7.6871 |  |  |  |  |  |
|  | 313.15 | 0.0032 | 1.66 | 7.4146 |  |  |  |  |  |
| β-CD | 303.15 | 0.0033 | 3.35 | 8.1167 | 5639.2 | -10.493 | -46.89 | -87.24 | -20.88 |
|  | 308.15 | 0.0032 | 2.42 | 7.7915 |  |  |  |  |  |
|  | 313.15 | 0.0032 | 1.85 | 7.5229 |  |  |  |  |  |

**Table S20**. Data of the van’t Hoff equation for calculation of thermodynamic
parameters ΔH^C0^, ΔS^C0^ and ΔG^C0^of different (PEH+α-CD) and (PEH+β-CD) inclusion complexes.

| **HOST** | **T(K^a^)** | **1/T** | **$\text{K}_{\text{a}}^{C0}$**  **(M^-1^×10^-3^)** | **ln$\text{K}_{\text{a}}^{C0}$** | **Slope** | **Intercept** | **ΔH^C0^**  **(KJ mol^-1^)** | **ΔS^C0^**  **(J mol^-1^ K^-1^)** | **ΔG^C0^**  **(KJ mol^-1^)** |
| --- | --- | --- | --- | --- | --- | --- | --- | --- | --- |
| α-CD | 303.15 | 0.0033 | 2.05 | 7.6256 | 3,754.3 | -4.7505 | -31.22 | -39.50 | -19.44 |
|  | 308.15 | 0.0032 | 1.72 | 7.4501 |  |  |  |  |  |
|  | 313.15 | 0.0032 | 1.38 | 7.2298 |  |  |  |  |  |
| β-CD | 303.15 | 0.0033 | 2.75 | 7.9194 | 4,679.3 | -7.5194 | -38.91 | -62.52 | -20.27 |
|  | 308.15 | 0.0032 | 2.12 | 7.6592 |  |  |  |  |  |
|  | 313.15 | 0.0032 | 1.68 | 7.4265 |  |  |  |  |  |

| **α-Cyclodextrin (400 MHz, Solvent: D_2_O), δ/ppm** | **β-Cyclodextrin (400 MHz, Solvent: D_2_O), δ/ppm** |
| --- | --- |
| 3.49-3.51 (6H, t, =8.00 Hz), 3.53-3.57 (6H, dd, J = 3.00, 10.00 Hz), 3.74-3.83 (18H, m), 3.87-3.91 (6H, t, J = 8Hz), 4.95-4.96 (6H, d, J = 4.00 Hz) | 3.49-3.54 (7H, t, J = 10.00 Hz), 3.56-3.60 (7H, dd, J = 9.6 Hz, 3.2 Hz), 3.79-3.85 (21H, m), 3.87-3.92 (7H, t, J = 9.2 Hz), 5.01-5.020 (7H, d, J = 3.6 Hz) |
| **SNP+α-CD inclusion complex (400 MHz, Solvent: D_2_O), δ/ppm)** | **SNP+β-CD inclusion complex (400 MHz, Solvent: D_2_O), δ/ppm)** |
| 2.439-2.447 (3H, d, J = 2.8 Hz), 2.887-2.981 (1H, m), 2.991-3.012 (2H, dd, J = 3.2, 8.4 Hz), 3.40-3.48 (12H, m), 3.686-3.743 (6H, m), 3.769-3.823 (18H, m), 4.894-4.893 (6H, d, J = 4.0 Hz), 6.617-6.649 (2H, d, J = 12.8 Hz), 7.05-7.079 (2H, d, J = 11.6 Hz) | 2.409-2.418 (3H, d, J = 3.6 Hz), 2.778-2.877 (1H, m), 2.891-2.922 (2H, dd, J = 5.6, 8.8 Hz), 3.38-3.49 (14H, m), 3.56-3.60 (7H, m), 3.65 3.77 (21H, m), 4.881-4.890 (7H, d, J = 3.6 Hz), 6.570-6.592 (2H, d, J = 8.8 Hz, 7.034-7.055 (2H, d, J = 8.4 Hz) |
| **PEH+α-CD inclusion complex (400 MHz, Solvent: D_2_O), δ/ppm)** | **PEH+β-CD inclusion complex (400 MHz, Solvent: D_2_O), δ/ppm)** |
| 2.594-2.602 (3H, d, J = 3.2 Hz), 3.086-3.139 (1H, m), 3.145-3.166 (2H, dd, J = 3.2, 8.4 Hz), 3.39-3.476 (12H, m), 3.680-3.735 (6H, m), 3.761-3.821 (18H, m), 4.83-4.88 (6H, m), 6.754-6.777 (2H, d, J = 9.2 Hz), 7.16-7.18 (2H, d, J = 8 Hz) | 2.633-2.641 (3H, d, J = 3.2 Hz), 3.015-3.070 (1H, m), 3.094-3.135 (2H, dd, J = 3.6, 12.8 Hz), 3.40-3.50 (14H, m), 3.61-3.635 (7H, m), 3.692-3.774 (21H, m), 4.929-4.895 (7H, m), 6.69-6.713 (2H, d, J = 9.2 Hz), 7.108-7.129 (2H, d, J = 8.4 Hz) |

**Table S21.** ^1^H NMR data of the pure α-Cyclodextrin, β-Cyclodextrin and the solid inclusion complexes.

**Table S22**. The observed peaks at different m/z with corresponding ions for the solid
inclusion complexes.

| SNP-α-CD inclusion complex | | PEH-α-CD inclusion complex | | SNP-β-CD inclusion complex | | PEH-β-CD inclusion complex | |
| --- | --- | --- | --- | --- | --- | --- | --- |
| **m/z** | **Ion** | **m/z** | **Ion** | **m/z** | **Ion** | **m/z** | **Ion** |
| 168.10 | [SNP+H]^+^ | 168.10 | [PEH+H]^+^ | 168.10 | [SNP+H]^+^ | 168.10 | [PEH+H]^+^ |
| 190.08 | [SNP+Na]^+^ | 190.08 | [PEH+Na]^+^ | 190.08 | [SNP+Na]^+^ | 190.08 | [PEH+Na]^+^ |
| 973.32 | [α-CD+H]^+^ | 973.32 | [α-CD+H]^+^ | 1135.38 | [β-CD+H]^+^ | 1135.38 | [β-CD+H]^+^ |
| 995.31 | [α-CD+Na]^+^ | 995.31 | [α-CD+Na]^+^ | 1157.36 | [β-CD+Na]^+^ | 1157.36 | [β-CD+Na]^+^ |
| 1140.42 | [SNP+α-CD+H]^+^ | 1140.42 | [PEH+α-CD+H]^+^ | 1302.47 | [SNP+β-CD+H]^+^ | 1302.47 | [PEH+β-CD+H]^+^ |
| 1162.40 | [SNP+α-CD+Na]^+^ | 1162.40 | [PEH+α-CD+Na]^+^ | 1324.45 | [SNP+β-CD+Na]^+^ | 1324.45 | [PEH+β-CD+Na]^+^ |

**Table S23.** Frequencies at FTIR spectra ofα-CD, β-CD, SNP, PEH and solid inclusion complexes.

| **α-cyclodextrin (α-CD)** | | **β-cyclodextrin (β-CD)** | |
| --- | --- | --- | --- |
| **Wavenumber (cm^-1^)** | **Group** | **Wavenumber (cm^-1^)** | **Group** |
| 3408.25 | -O-H stretching | 3370.21 | -O-H stretching |
| 2932.12 | -C-H stretching | 2916.35 | -C-H stretching |
| 1406.17 | -C-H and -O-H bending | 1412.27 | -C-H and -O-H bending |
| 1154.26 | C-O-C bending | 1158.14 | C-O-C bending |
| 1030.19 | C-C-O stretching | 1026.52 | C-C-O stretching |
| 978.23 | skeletal vibration involving α-1,4linkage | 938.08 | skeletal vibration involving α-1,4linkage |

| **Synephrine (SNP)** | | **Phenylephrine hydrochloride (PEH)** | |
| --- | --- | --- | --- |
| **Wavenumber (cm^-1^)** | **Group** | **Wavenumber (cm^-1^)** | **Group** |
| 3288.34 | -O-H stretching/N-H stretching | 3396.45 | -O-H stretching/N-H stretching |
| 2878.21 | -C-H stretching | 2806.31 | -C-H stretching |
| 1610.31 | C=C stretching | 1602.37 | C=C stretching |
| 1508.17 | Aromatic C=C bending/N-H bending | 1458.51 | -C-H bending (methyl/methylene) |
| 1264.11 | -C-O stretching (phenol) | 1272.59 | -C-O stretching (phenol) |
| 1096.11 | -C-N stretching | 1172.23 | -C-N stretching |
| 1054.12 | -C-O stretching (secondary alcohol) | 1070.13 | -C-O stretching (secondary alcohol) |
| 782.42 | Aromatic -C-H out-of-plane bending | 792.26 | Aromatic -C-H out-of-plane bending |
| 640.37 | Aromatic -C-H out-of-plane bending | 700.15 | Aromatic -C-H out-of-plane bending |
| **SNP+α-CD** | | **SNP+β-CD** | |
| **Wavenumber (cm^-1^)** | **Group** | **Wavenumber (cm^-1^)** | **Group** |
| 3374.08 | -O-H stretching/N-H stretching | 3320.19 | -O-H stretching/N-H stretching |
| 2929.29 | -C-H stretching | 2932.18 | -C-H stretching |
| 1628.35 | C=C stretching | 1604.08 | C=C stretching |
| 1333.45 | -C-N stretching | 1336.35 | -C-N stretching |
| 1154.27 | -C-O stretching (phenol) | 1158.08 | -C-O stretching (phenol) |
| 1030.23 | -C-O stretching (secondary alcohol) | 1032.29 | -C-O stretching (secondary alcohol) |
| 707.25 | Aromatic -C-H out-of-plane bending | 754.36 | Aromatic -C-H out-of-plane bending |
| 583.26 | Aromatic -C-H out-of-plane bending | 582.13 | Aromatic -C-H out-of-plane bending |
| **PEH+α-CD** | | **PEH+β-CD** | |
| **Wavenumber (cm^-1^)** | **Group** | **Wavenumber (cm^-1^)** | **Group** |
| 3370.14 | -O-H stretching/N-H stretching | 3356.19 | -O-H stretching/N-H stretching |
| 2930.31 | -C-H stretching | 2926.32 | -C-H stretching |
| 1616.21 | C=C stretching | 1618.14 | C=C stretching |
| 1398.21 | -C-N stretching | 1370.35 | -C-N stretching |
| 1152.24 | -C-O stretching (phenol) | 1156.26 | -C-O stretching (phenol) |
| 1029.13 | -C-O stretching (secondary alcohol) | 1030.31 | -C-O stretching (secondary alcohol) |
| 716.19 | Aromatic -C-H out-of-plane bending | 756.39 | Aromatic -C-H out-of-plane bending |
| 705.20 | Aromatic -C-H out-of-plane bending | 685.11 | Aromatic -C-H out-of-plane bending |

**FIGURES**:

**Figure S1** Benesi-Hildebrand double reciprocal plots for the effect of *α* and β-CD on the
absorbance of SNP (219 nm) at different temperatures.

**Figure S2** Benesi-Hildebrand double reciprocal plots for the effect of *α* and β-CD on the
absorbance of PEH (209 nm) at different temperatures.

**Figure S3 (a,b)** Spectro-fluorimetric spectra for the generation of Benesi-Hildebrand double reciprocal plot of (SNP+α-CD) and (SNP+β-CD) systems at, λ_max_ = 301 (nm) and 298.15 K.

**Figure S4 (a,b)** Spectro-fluorimetric spectra for the generation of Benesi-Hildebrand double reciprocal plot of (PEH+α-CD) and (PEH+β-CD) systems at, λ_max_ = 301 (nm) and 298.15 K.

**Figure S5 (a,b)**Benesi-Hildebrand double reciprocal plots for the effect of (a) *α*-CD and (b) β-CD on the emission of SNP at, λ_max_ = 301 nm and 298.15 K.

**Figure S6 (a,b)**Benesi-Hildebrand double reciprocal plots for the effect of (a) *α*-CD and (b) β-CD on the emission of PEH at, λ_max_ = 301 nm and 298.15 K.

**Figure S7** Plot of lnKa vs 1/T for the interaction of SNP with *α*-CD (■) and β-CD (●).

**Figure S8** Plot of lnKa vs 1/T for the interaction of PEH with *α*-CD (●) and β-CD (■).

**Figure S9** Plot of ln$\text{K}_{\text{a}}^{\text{θ}}$vs 1/T for the interaction of SNP with *α*-CD (■) and β-CD (●).

**Figure S10**. Plot of ln$\text{K}_{\text{a}}^{\text{θ}}$vs 1/T for the interaction of PEH with *α*-CD (●) and β-CD (■).

**Figure S11** Plot of ln$\text{K}_{\text{a}}^{\text{C}}$vs 1/T for the interaction of SNP with *α*-CD (■) and β-CD (●).

**Figure S12** Plot of ln$\text{K}_{\text{a}}^{\text{C}}$vs 1/T for the interaction of PEH with *α*-CD (●) and β-CD (■).


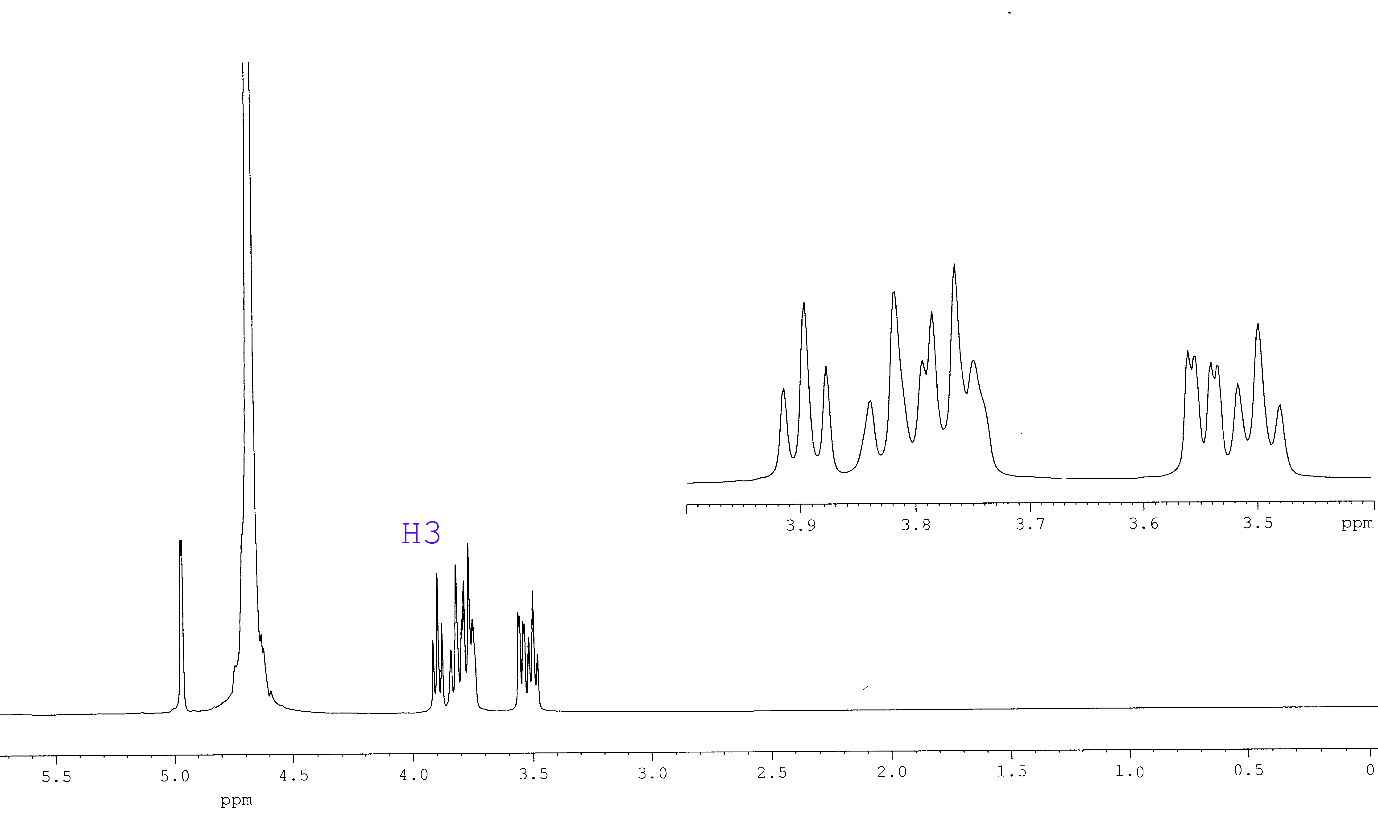


**Figure S13** ^1^H NMR spectra of pure α-CD.

**
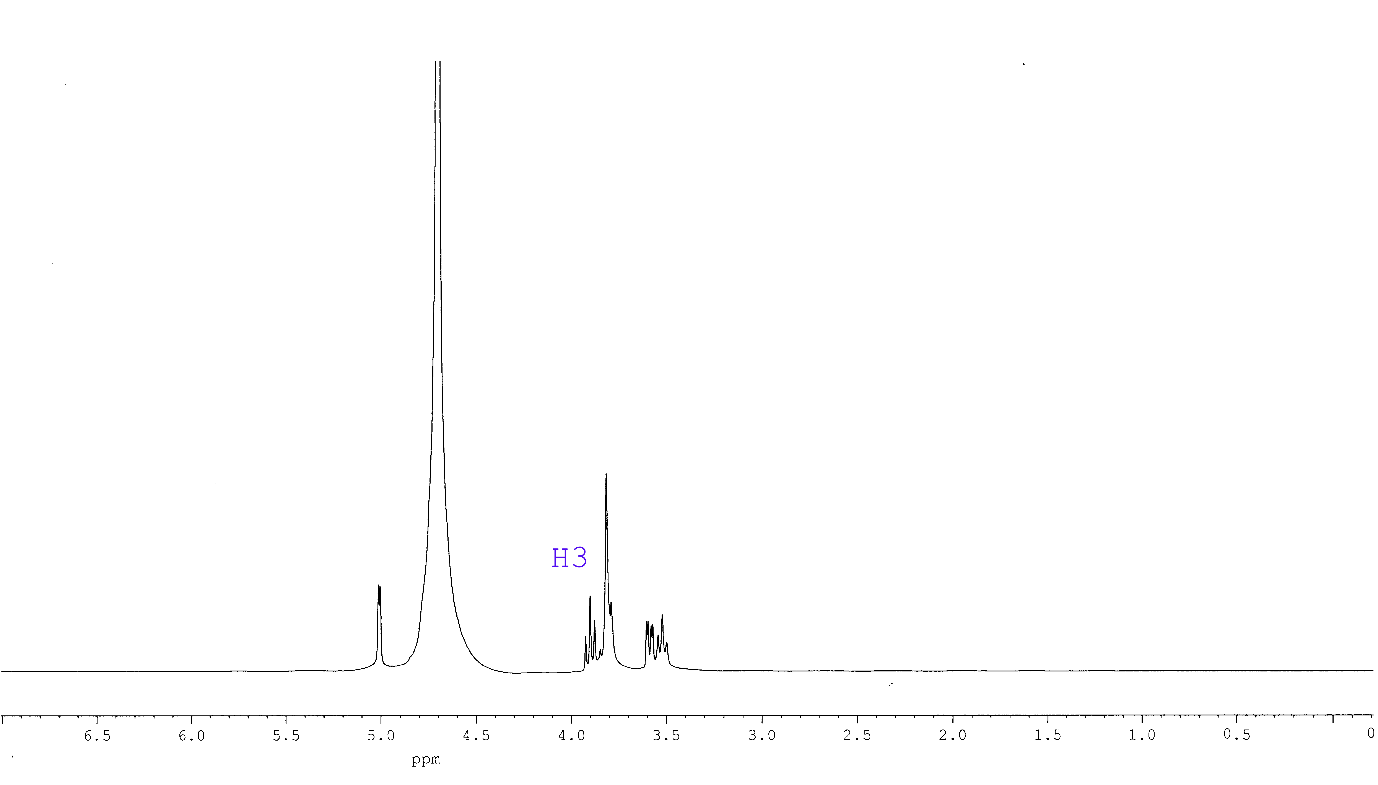
**

**Figure S14** ^1^H NMR spectra of pure β-CD.


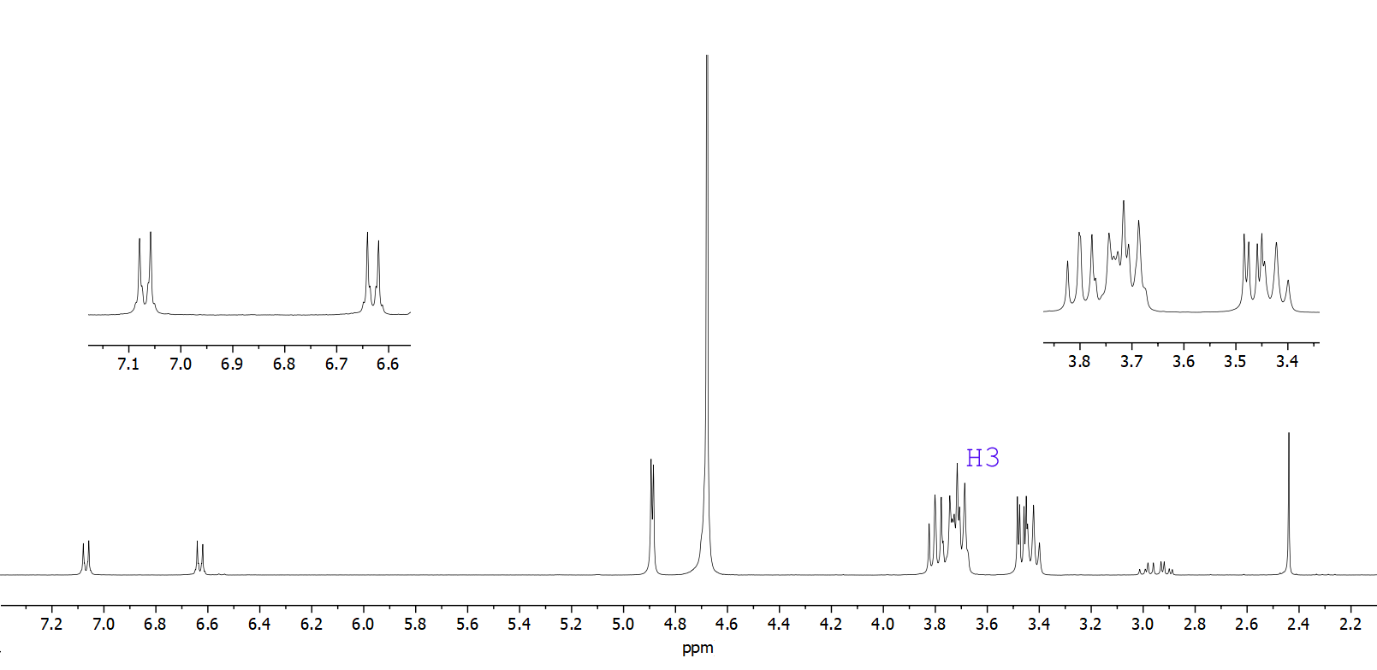


**Figure S15** ^1^H NMR spectra of (SNP+α-CD) inclusion complex.


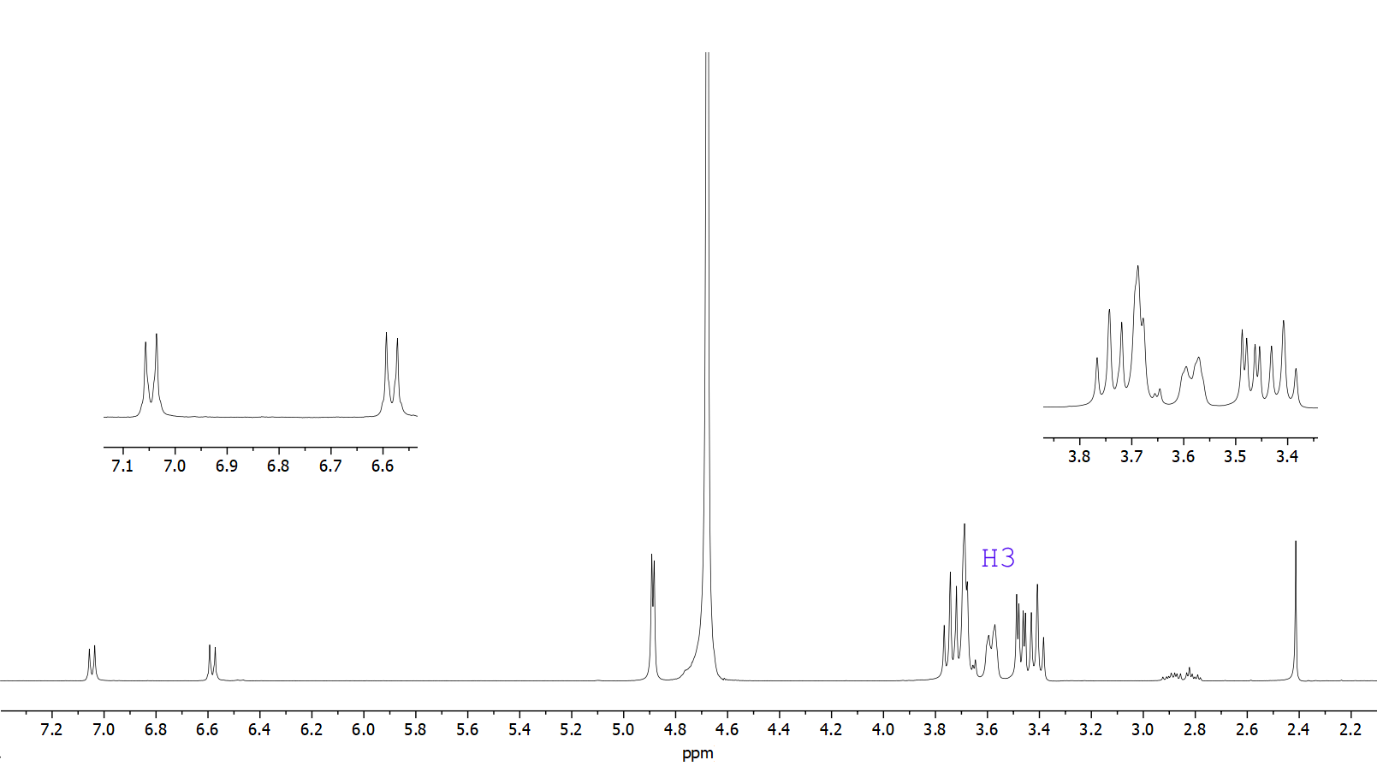


**Figure S16** ^1^H NMR spectra of (SNP+β-CD) inclusion complex.


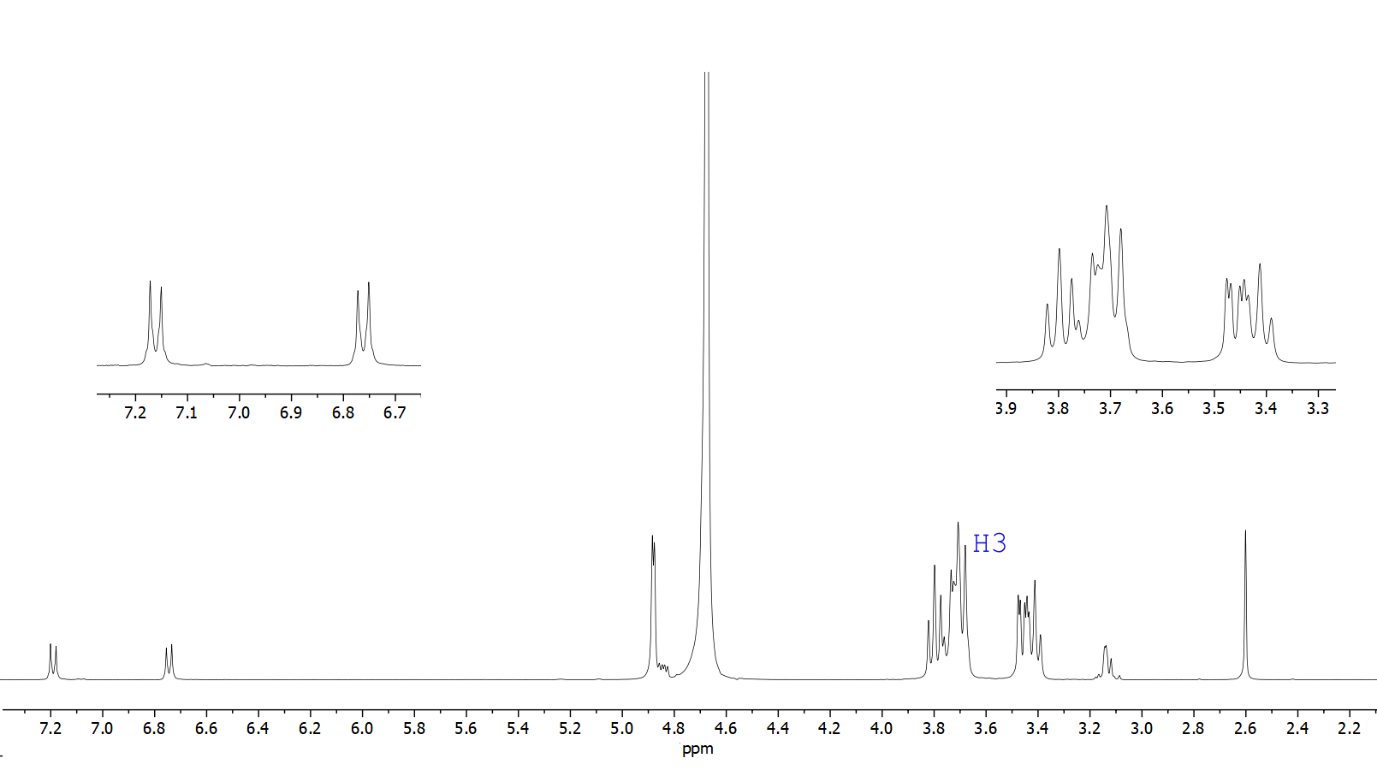


**Figure S17** ^1^H NMR spectra of (PEH+α-CD) inclusion complex.


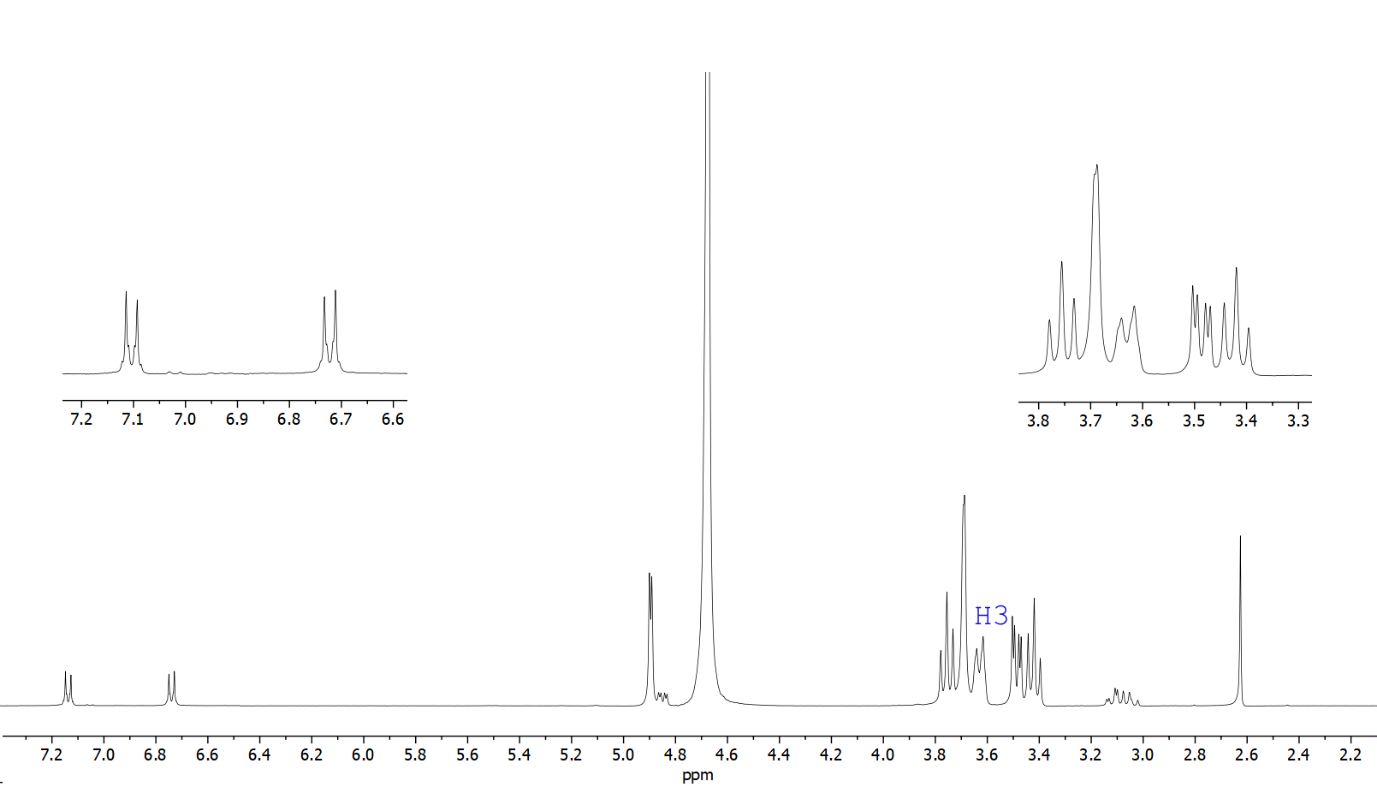


**Figure S18** ^1^H NMR spectra of (PEH+β-CD) inclusion complex.
